# Controlled grafting of vinylic monomers on polyolefins: a robust mathematical modeling approach

**DOI:** 10.1080/15685551.2016.1239166

**Published:** 2016-10-25

**Authors:** Mohammad Reza Saeb, Babak Rezaee, Alireza Shadman, Krzysztof Formela, Zahed Ahmadi, Farkhondeh Hemmati, Tayebeh Sadat Kermaniyan, Yousef Mohammadi

**Affiliations:** ^a^ Department of Resin and Additives, Institute for Color Science and Technology, Tehran, Iran; ^b^ Department of Industrial Engineering, Ferdowsi University of Mashhad, Mashhad, Iran; ^c^ Faculty of Chemistry, Department of Polymer Technology, Gdansk University of Technology, Gdansk, Poland; ^d^ Color and Polymer Research Center, Amirkabir University of Technology, Tehran, Iran; ^e^ Department of Polymer Engineering and Color Technology, Amirkabir University of Technology, Tehran, Iran; ^f^ Petrochemical Research and Technology Company (NPC-rt), National Petrochemical Company (NPC), Tehran, Iran

**Keywords:** Free-radical grafting, glycidyl methacrylate, modeling, artificial intelligence, optimization, desirability function

## Abstract

Experimental and mathematical modeling analyses were used for controlling melt free-radical grafting of vinylic monomers on polyolefins and, thereby, reducing the disturbance of undesired cross-linking of polyolefins. Response surface, desirability function, and artificial intelligence methodologies were blended to modeling/optimization of grafting reaction in terms of vinylic monomer content, peroxide initiator concentration, and melt-processing time. An in-house code was developed based on artificial neural network that learns and mimics processing torque and grafting of glycidyl methacrylate (GMA) typical vinylic monomer on high-density polyethylene (HDPE). Application of response surface and desirability function enabled concurrent optimization of processing torque and GMA grafting on HDPE, through which we quantified for the first time competition between parallel reactions taking place during melt processing: (i) desirable grafting of GMA on HDPE; (ii) undesirable cross-linking of HDPE. The proposed robust mathematical modeling approach can precisely learn the behavior of grafting reaction of vinylic monomers on polyolefins and be placed into practice in finding exact operating condition needed for efficient grafting of reactive monomers on polyolefins.

## Highlights

• Exact modeling of melt functionalization of polyethylene with vinylic monomers.
• Proposing an intelligent robust mathematical model for controlled grafting reaction.
• Verification of model predictions against measured data of GMA grafting on HDPE.
• Multi-objective optimization of processing torque and grafting ratio of GMA on HDPE.
• Picturing the competitive antagonism of cross-linking by the grafting reaction.

## 1. Introduction

Polyolefins (POs) such as polyethylene (PE) and polypropylene (PP) have received an increasing demand among thermoplastic family of polymers thanks to their comparatively low price and reasonable mechanical and processing properties.[1–3] The lack of polar groups in the POs, however, entails some obvious shortcomings including poor adhesion, low paintability, and poor compatibility with polar polymers.[4,5] Theoretical and experimental studies confirmed that morphological, mechanical, rheological, and thermal properties of PO-based blends can be improved by material and processing parameters.[6–11] It was also shown that the use of appropriate compatibilizer precursors enhances degree of interfacial adhesion in immiscible polymer blends, thereby enhances ultimate properties.[12–16] The challenging point still is finding a proper precursor which delivers sufficient interfacial adhesion and fulfills the requirements of practical applications.

A widely known chemical functionalization method is the attachment of polar monomers to POs via melt free-radical polymerization. The resulting compatibilizers can then be used to connect the polymers of dissimilar polarities in the molten state.[17,18] Reactive extrusion aided by incorporation of a free-radical initiator into the mixture of polar monomers and PO was found to be a helpful technique for the attachment of functional groups to PO chains.[16,18–20] In parallel, numerous studies have focused on using laboratory-scale internal mixers to attach functional groups to the POs in the melt state.[18,20–22]

Many reactive vinylic monomers among which are maleic anhydride (MA) and glycidyl methacrylate (GMA) have been attached to various polymers through reactive melt processing to attain PO-grafted macromonomers (MM), hereafter referred to as PO-*g*-MM.[23] The presence of unsaturated groups in GMA structure enables free-radical grafting reaction, while the presence of epoxy groups facilitates strong interactions with a variety of functional groups including hydroxyls, amines, and anhydrides.[19,23] Accordingly, different thermoplastic polymers such as polycaprolactone, polypropylene, low- and high-density PE (HDPE) have been functionalized with GMA monomer via melt free-radical grafting.[20,24,25] Studies reveal that, even in the presence of an excess amount of peroxide, the conversion of GMA grafting reaction on PP does not exceed 10%.[26] Such low grafting level suggests that the reactivity of GMA could be deteriorated during free-radical grafting. The grafting efficiency is controlled by many factors, among which is the time of melt mixing. The grafting of GMA on polymers undergoes an early-stage upward trend followed by a downward slope, which arises from the fact that depolymerization of GMA-bearing polymer chains is possible during the free-radical grafting time span.[22] This situation could be resolved by addition of a comonomer, e.g. styrene, but the molar ratio of the comonomer to GMA has to be optimized to ensure an efficient grafting reaction.[22,27–29] Additionally, the unfavorable cross-linking reaction, which takes place in parallel with grafting of PE, disturbs the main grafting reaction. Such complexities originate from the dynamic interactive nature of variables during the reaction. Thus, to uncover the contribution of aforementioned competitive reactions to increment of mixing torque, we need an advanced tool with the ability of learning nonlinear dynamic behavior of process and generalizing the learned pattern to design several other grafting and cross-linking reactions. We have successfully applied Taguchi [26,30–32] and response surface methodologies (RSM) [33–36] to determine the individual and interactive effects of changing variables when confronting such critical situations. These techniques work on the basis of fitting first- and second-order interpolating functions and cannot solely predict peculiar trends due to the lack of ability of learning the behavior of the process.

In a previous work, we used RSM to study the effects of material and processing parameters so as to predict efficiency of GMA grafting on HDPE in an internal mixer.[14] Application of RSM enabled design of experiments and proposed a quadratic model to the input variables (dicumyl peroxide content, GMA content and reaction time) and processing torque values. The second-order model derived using RSM could predict the final torque reasonably well. We also measured the melt flow rate of some specimens to give some insight into undesirable cross-linking of HDPE in the presence of DCP. However, the adequacy and reliability of regression as well as the contribution of grafting to the value of final processing torque could not be monitored on account of inability of RSM to account for learning intricate nonlinear behavior of grafting and cross-linking reactions.[20]

To present a direct picture of competitive antagonism of cross-linking of HDPE by the GMA grafting on HDPE, we propose in this work hybridization of RSM with other stochastic modeling approaches. We designed and prepared different samples and determined ratio of GMA grafting on HDPE by titration and Fourier transform infrared (FTIR) measurements. In the first step of modeling, we used analysis of variance (ANOVA) to assess the reliability of RSM modeling with a more detailed statistical analysis compared to our previous work. Different models including linear and partial (or full) quadratic were examined to obtain the best approximating function over the range of variables. This provided a rough estimation of the contribution of GMA grafting reaction to the amount of final processing torque. Then, we concurrently optimized grafting efficiency and final processing torque responses. Since we knew that the value of the processing torque would depend on both grafting and cross-linking reactions, adequacy and reliability of the models were compared using different statistical criteria to the experimental data. To precisely capture trends in experimental data, what we could limitedly detect by RSM, we developed a computer code based on artificial neural network (ANN) and genetic algorithm (GA) approaches. The possibility of training the ANN model with numerous trial and errors could principally guarantee a higher reliability and help for understanding the interactive effects between changing variables, which is different from what one would expect from RSM. The ability to test validity of experiments not used for training was another advantageous feature to the GA-based ANN modeling approach. Hybridizing the capabilities of RSM, ANN, and GA approaches, we provide for the first time a pattern on the competition between grafting reaction of GMA on HDPE and cross-linking of HDPE in the presence of DCP. This mathematical framework can be applied in melt production of PO-*g*-MM compatibilizers of similar families.

## 2. Experimental

### 2.1. Materials and characterization

HDPE under trade name of HTA108 was purchased from ExxonMobil Chemical Co. (USA) with density of 0.961 g cm^−3^ and melt flow index of 0.7 g.10 min^−1^ (190 °C; 2.16 kg). GMA (97% purity) and styrene (99% purity) monomers, respectively having density values of 1.08 and 0.906 g cm^−3^, as well as other chemicals used in titration and back titration were all provided by Merck Co. (Germany) and used without further purification.

**Scheme 1.**
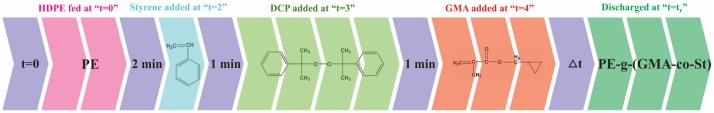
Time schedule applied in melt grafting of GMA on HDPE.

Melt free-radical grafting of GMA on HDPE was carried out using an internal Brabender Plasticorder PL2200 mixing machine. HDPE was first introduced into the reaction chamber allowed to become fully molten at 180 °C and rotor speed of 60 rpm. After 2 minutes , a homogeneous melt was reached and styrene comonomer was added using a syringe needle, then GMA and DCP were added at specified times, as schematically shown in Scheme 1. Injections were performed very meticulously by a syringe to the bulk of molten HDPE to prevent evaporation of styrene.

Since the reaction might be continued even after the chamber was discharged from molten polymer, the products were immediately immersed in zero-degree-water/ice mixture to enable accurate monitoring of reaction time effect on grafting yield. The purified HDPE-*g*-GMA samples were filtered, washed with excess acetone and dried under a vacuum at 80 °C for 10 hours , and compression molded at 300 bar pressure and 180°C into films 0.5 mm in thickness and used for FTIR analysis. FTIR measurements were conducted on a JASCO FTIR-6300 (Japan) with resolution of 4 cm^−1^ for 32 scans in the wavelength range of 4000–400 cm^−1^.

### 2.2. Calculation of grafting ratio by FTIR and titration methods

Samples obtained from melt processing were dissolved in hot xylene (100 ml) and stirred for about 2 hours  and substantially precipitated with excess amount of acetone (200 ml). Prior to precipitation, the hot solution of polymer was filtered through a fine grid to separate gels due to cross-linking of HDPE chains in the presence of DCP. The precipitated sample was filtered, washed three times with excess acetone and dried under vacuum at 80 °C overnight for complete separation of unreacted monomers, homo- and co-polymer of GMA and styrene.[20,22] Approximately 1 g of the purified sample was dissolved in 150 ml of hot xylene followed by addition of 1.5 ml of 0.3 M xylene solution in trichloro acetic acid (TCA). The mixture was maintained at 110 °C for 2 hours  so as to complete the reaction of TCA with grafted GMA. The resulting solution was precipitated with amount of excess acetone (300 ml), filtered and washed two times. The filtrate was titrated with 0.1 M KOH solution in methanol and then was back-titrated with 0.1 M HCl solution in isopropanol using phenolphthalein as an indicator.[20] The ratio of GMA grafting was determined by constructing a calibration curve correlating FTIR and titration results. For grafted samples, a peak appears in FTIR spectrum at around 1730 cm^−1^ which is attributed to carbonyl stretching of grafted GMA, while peak at 1368 cm^−1^ corresponds to the HDPE methylene group stretching. The peak detected nearby 720 cm^−1^ stands for methylene group of styrene. The ratio of peak intensities of GMA carbonyl to HDPE methylene group (I_1730_/I_1368_) was correlated to absolute GMA grafting yield measured by titration method.[20] Four different samples with different initial GMA content were prepared to construct calibration curve.

## 3. Model development

### 3.1. Experimental design by RSM

To detect efficiency of GMA grafting and processing torque, three input variables including DCP content $\left(x_{1}\right)$, GMA content $\left(x_{2}\right)$, and reaction time $\left(x_{3}\right)$ are chosen and an experimental design based on RSM was put into practice. The output responses were the final torque (*y*_1_) applied to the sample at the end of melting process, as recorded by the machine, and the grafting level (*y*_2_) measured experimentally based on spectroscopic and titration techniques.[20] Samples used to construct calibration curve were prepared through a simple protocol with DCP content of 0.25 phr and variable GMA contents of 2, 4, 6, and 8 phr for reactions completed in 6 minutes . A three-factor-ﬁve-level central composite design (CCD) was used to design experimental runs for statistical analyses. It is well-documented that a CCD with *k* factors requires *n*_*F*_ factorial runs (for full two-level factorial designs: *n*_*F*_ = 2^*k*^), 2*k* axial runs, and *n*_*C*_ center points (usually between 3 to 5 to guarantee a good prediction of the response). Accordingly, the three-factor-five-level CCD method was applied ending in 20 experimental runs. More details on RSM method can be found in the Supporting Materials (SM). Table 1 presents the architecture and coded levels of the chosen factors. Design Expert software package version 7 was used for design analysis and optimization.

**Table 1.** Architecture, ranges and coded levels of independent variables.

Variables	Symbols	Range and levels				
		-1.68	-1	0	+1	+1.68
Reaction time (sec)	*x*_1_	180	240	330	420	+480
DCP content (phr)	*x*_2_	0.20	0.28	0.40	0.52	0.6
GMA content (phr)	*x*_3_	3.00	4.22	6.00	7.78	9.00

### 3.2. Artificial intelligence-based modeling

In the current study, GA-based ANN model was developed and put into practice to capture and optimize melt free-radical functionalization of polyolefins with vinylic monomers. The model enabled identifying the intricate relationship between the input and output variables through a stochastic computational modeling approach. When ANN has been structured for a particular application, it is ready to be trained. This training allows the network to learn the appropriate behavior for the defined task. During the training phase, the best weights and biases of the network were found to minimize the prediction error made by the network. The error of a particular configuration of the network can be determined by running all the training cases through the network, comparing the actual outputs generated by the model with the desired (target) outputs. When the training is completed, the ANN has gained capability of predicting the output upon receiving any input similar to the pattern it was taught. Some basic concepts on ANN modeling are provided in SM.

Target variables including final processing torque and GMA grafting level were recorded under the experimental circumstances proposed by the RSM (Table 2). The term ‘scenario’ refers to experimental runs numbered in accord with the order proposed by the RSM. After feeding all scenarios to the ANN model, they will be tested until the model finds the situation of experiments to satisfy the defined error for training and test phases. Fifteen scenarios were randomly selected and fed into the ANN model to recognize the required experimental runs. The ANN considers such runs as ‘experiment’ and sorts them according to satisfactoriness order.

**Table 2.** Different scenarios considered as input data to be fed to the neural network model.

Scenario	1st Input (*x*_1_) reaction time (s)	2nd Input (*x*_2_) DCP (phr)	3rd Input (*x*_3_) GMA (phr)	1st Output (*y*_1_) final torque (Nm)	2nd Output (*y*_2_) GMA grafting level (wt.%)
1	330	0.40	6.00	50.40	3.194
2	240	0.28	4.22	44.52	3.314
3	240	0.52	7.78	56.24	2.999
4	330	0.6	6.00	49.84	3.036
5	330	0.40	6.00	43.79	3.073
6	330	0.20	6.00	37.01	2.368
7	330	0.40	9.00	48.42	3.268
8	420	0.52	4.22	43.52	2.526
9	180	0.40	6.00	26.88	2.516
10	240	0.52	4.22	56.74	2.73
11	240	0.28	7.78	57.35	3.426
12	330	0.40	3.00	46.34	2.098
13	330	0.40	6.00	46.27	2.767
14	330	0.40	6.00	46.73	2.897
15	330	0.40	6.00	47.20	2.813
16	420	0.28	7.78	40.58	3.259
17	330	0.40	6.00	46.11	2.934
18	480	0.40	6.00	36.33	3.686
19	420	0.28	4.22	35.07	2.006
20	420	0.52	7.78	47.12	3.798

Prior to feeding the data to the ANN model, it is essential to normalize the input and response variables. This prevents large numbers from overriding small ones and consequently prevents premature saturation of hidden nodes. The hyperbolic tangent sigmoid activation function has been used in this regard to produce normalized quantities scattered in the span of −1 to +1.

The input and target quantities normalized in the assigned interval are generated according to the following function:

(1) $$X_{i}=2\times \left[\frac{x_{i}-x_{\min}}{x_{\min}-x_{\max}}\right]-1$$

where *X*_*i*_ is the normalized value of the input variable of *x*_*i*_, and *x*_min_ and *x*_max_ are respectively the minimum and maximum values of that variable or target functions.

The normalized values corresponding to the experimental data are summarized in Table S1 in SM. The normalized data were subsequently treated into two different sets as training and test datasets. Accordingly, 80% of them (16 experiments) were randomly selected and fed into the model for training, while the remainder, i.e. 20% (4 experiments), were considered to test the developed ANN model. This procedure was repeated until the desired accuracy has been attained on account of the best ‘experiment’ arrangement. Tables S2 and S3 in SM summarize data chosen for training and testing of ANN model, respectively.

The number of neurons in the input and output layers is governed by the dimensionality of the problem. It has been proven that two hidden layers are sufficient to approximate any function to an arbitrary order of accuracy [37] and one hidden layer would be adequate to approximate a bounded continuous function to an arbitrary accuracy.[38] Basically, there is no systematic procedure for determining the number of hidden units before modeling, although higher accuracies are often the result of a larger search space, but cause overfitting.

Yet a little is known for systematic calculation of number of neurons in the hidden layers.[39,40] The number of neurons or processing elements in the hidden layer can usually be selected by considering the number of data points available for training the network as well as the complexity of the relationship between the input and the output parameters.[41] Typically, the trial and error method is used to construct the most desirable model.

**Figure 1.**
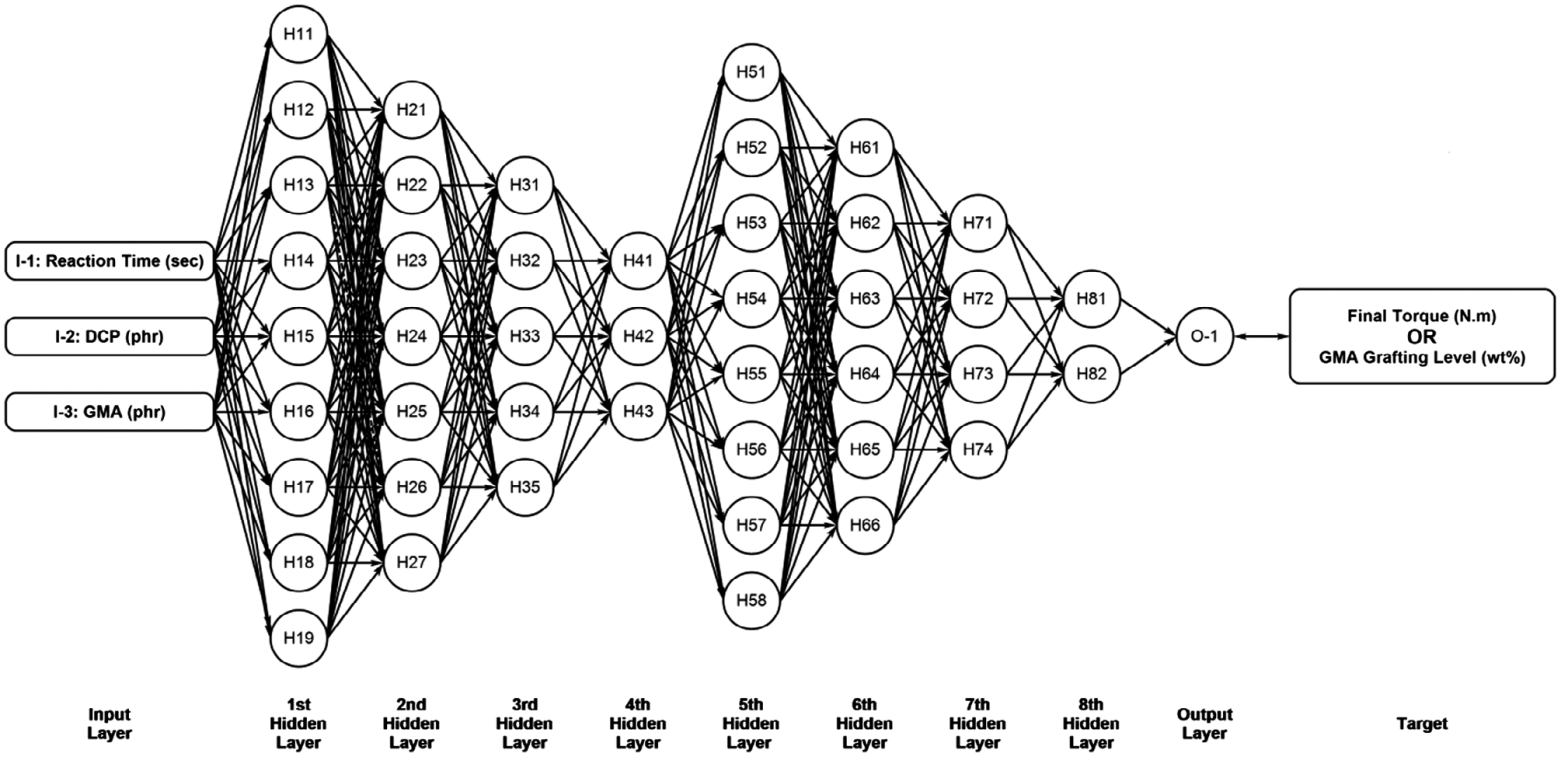
Architecture of ANN model used for prediction of final torque and grafting level.

For modeling the target functions of this work, we used a nine-layer ANN having architecture of 9-7-5-3-8-6-4-2-1, in which the first to eights hidden layers possess 9, 7, 5, 3, 8, 6, 4, and 2 neurons, respectively (Figure 1). Accordingly, two ANNs were developed to predict final torque and grafting level separately.

The hyperbolic tangent sigmoid function was used as activation transfer function:

(2) $$f\left(x\right)=\frac{e^{x}-e^{-x}}{e^{x}+e^{-x}}$$

The ANN models were trained by the first set of data through which the biases and weights of inter-connections between the neurons were systematically manipulated until the desired error level was achieved. The model was subsequently tested by feeding the second set of data into the network (Table S3).

**Figure 2.**
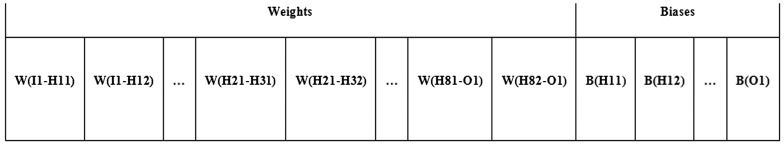
Defined chromosome structure for coding the unknown neural network parameters (weights and biases are represented by W and B, respectively).

For training the defined ANN as well as optimization and determination of the unknown parameters of the network, we used the evolutionary approach of GA. In this regard, unknown parameters of the network including the weights and biases are coded as a chromosome. Since it is customary to define a bias for any neuron in the hidden and output layers of the ANN, the number of unknown biases is equal to that of neurons. Accordingly, the number of unknown biases and unknown weights are 45 and 246, respectively. As in Figure 2, unknown parameters of the network are 291, which are coded as a chromosome composed of 291 components or genes. Also, the initial 246 genes in this chromosome are representative of the unknown weights of the ANN, while the remainders of 45 are those dedicated to unknown biases, which are placed correspondingly one after another from the left to the right hand in the defined chromosome.

It is to be noted that each gene situated in the chromosome structure can take values in the range of −1 to +1. To optimize parameters of the model, a population of 50 chromosomes is randomly generated and the information of each chromosome, e.g. chromosome number *j*, is independently entered to the predefined ANN structure, so that, the network has been evaluated based upon weights and biases verbalized by the assigned chromosome. Therefore, all normalized input values summarized in Table S1 are fed into the network to calculate the specified output. The reliability of ANN model has then been checked in terms of mean of squared error (MSE):

(3) $$\text{MSE}\left(j\right)=\frac{1}{N_{\text{training}}}\sum_{i=1}^{N_{\text{training}}}{\left(x_{i,\text{ANN}}\left(j\right)-x_{i,\text{Target}}\right)}^{2}$$

where *N*_training_ is the number of data used for training (16 scenarios) and $\text{MSE}\left(j\right)$ is the MSE for the *j*th chromosome. In addition, $x_{i,\text{ANN}}\left(j\right)$ is the output of the ANN corresponding to the *i*th scenario with respect to the *j*th chromosome *j*, and *x*_*i*,Target_ is the quantity of target function in regard with *i*th scenario. Also, the corresponding error of the network can be calculated as follows:

(4) $$\text{Error}=\left[\frac{\sqrt{\text{MSE}}}{\text{Max}\left(\text{Error}\right)}\right]\times 100=\left[\frac{\sqrt{\text{MSE}}}{2}\right]\times 100$$

where $\text{Max}\left(\text{Error}\right)$ is the maximum expectable error of the network, which takes the value of 2 considering the fact that all data are normalized between −1 and +1.

After error for each chromosome was determined through minimizing the MSE, this criterion was served to sort chromosomes from the smaller to the bigger; thereby the best chromosome was identified. Next, selection, mating, crossover, and mutation operators were applied to the population and the optimization process continued until the desired chromosome was reached in accord with specified target. Selection operator we recognized by merging, sorting, and truncating mechanisms. The mating operator couples the remainder of chromosomes in accord with roulette wheel selection mechanism.[42] For the sake of mating, one-point recombination was employed to enable crossover of two parent chromosomes to create child chromosomes. Ultimately, the mutation operator picked randomly one gene from the selected chromosome and exchanged stochastically its quantity with a new digit in the range of −1 to +1. The mutation rate was set to 20%, means that in each iteration or epoch, the mutation operator was applied to 20% of the child chromosomes. The quantities of parameters used for evolutionary optimization of ANN based upon GA methodology are given in Table 3.

**Table 3.** Value of parameters related to neural network optimization based on developed GA.

Value	Optimization parameter
50	Initial population size
Merge, sort, and truncate	Selection mechanism
Roulette wheel selection	Mating mechanism
Single-point crossover	Crossover mechanism
20%	Mutation rate

Once the training process has been completed, means that the MSE assigned to the best chromosome reached the possible minimum level (6.4 × 10^−3^ or 4%), all data in Table S3 were normalized and fed consecutively to the ANN. The weights and biases were accordingly adjusted to satisfy error criterion assigned to test phase. The maximum error in test procedure was set to 20%, equal to the MSE of 1.6 × 10^−1^.

**Figure 3.**
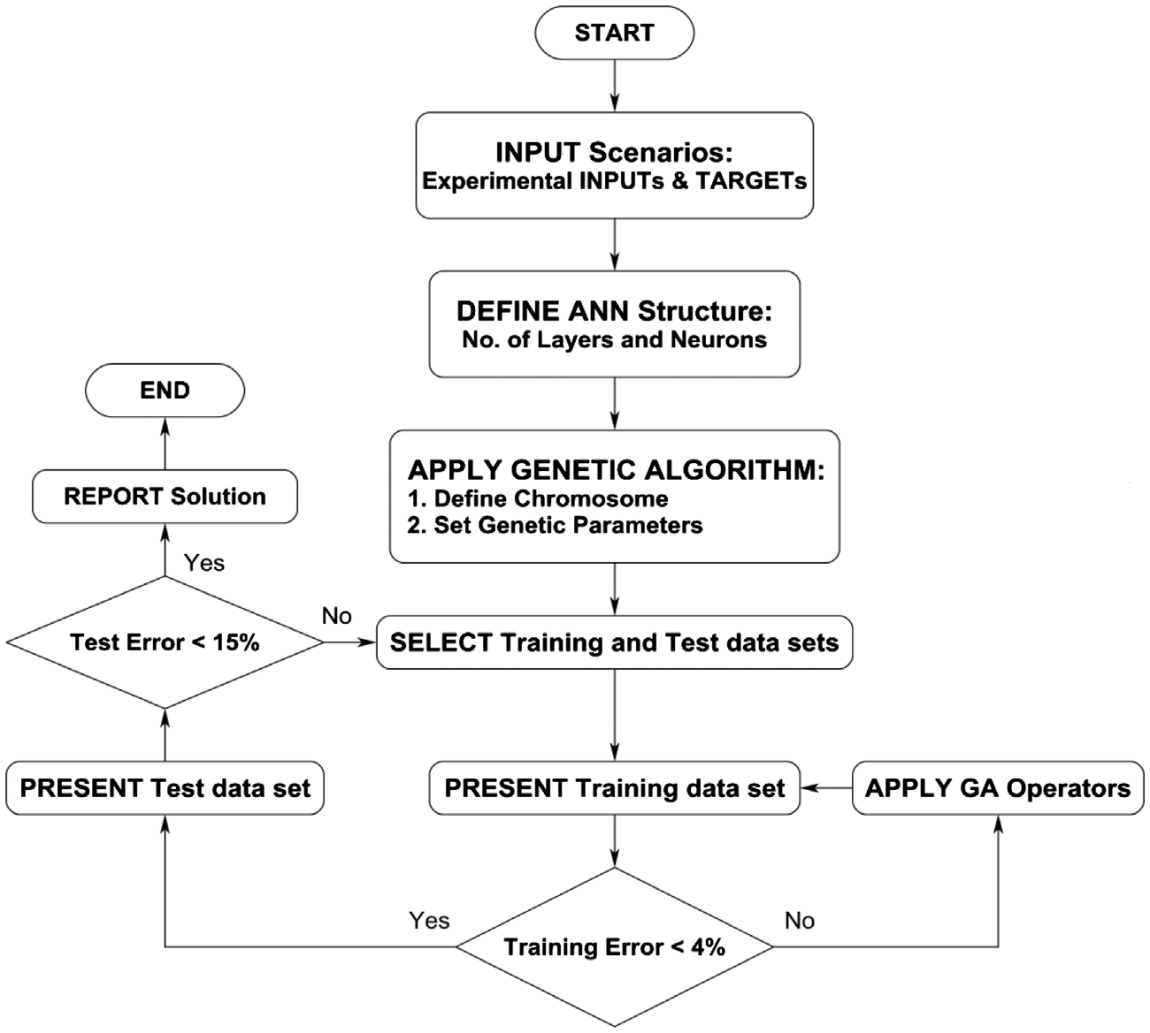
Flowchart of optimization process and determination of unknown parameters for the neural network.

According to flowchart demonstrated in Figure 3, a well-organized computer code was written in PASCAL programming language (Lazarus IDE) and compiled into 64-bits executable using FPC 2.6.2. Modeling was performed on a desktop computer with Intel Core i7-3770 K (3.50 GHz), 32 GB of memory (2133 MHz), under Windows 7 Ultimate 64-bit operating system.

**Figure 4.**
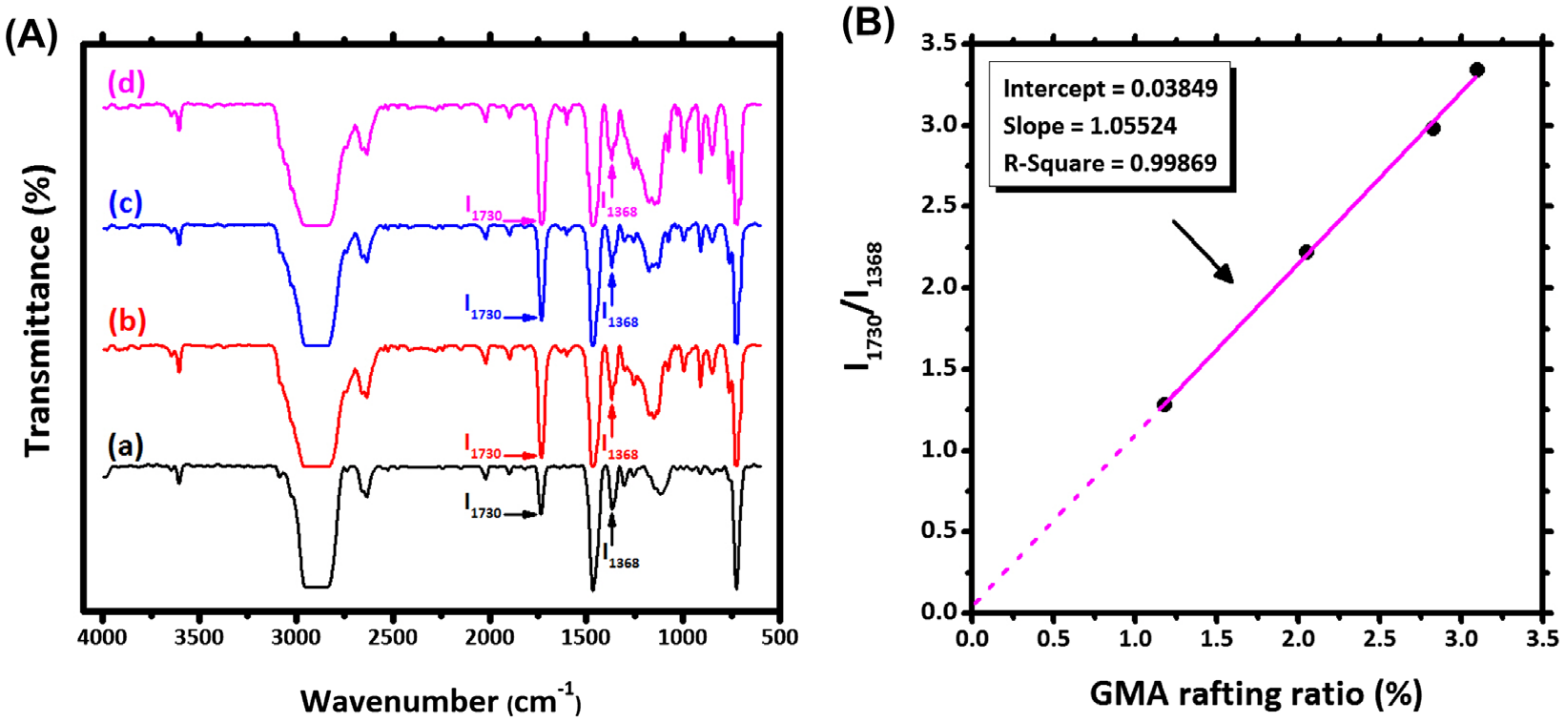
FTIR spectra of samples with DCP content of 0.25 phr and variable GMA contents of 2, 4, 6, and 8 phr specimen (A), and FTIR calibration curve of GMA grafting on HDPE (B).

**Figure 5.**
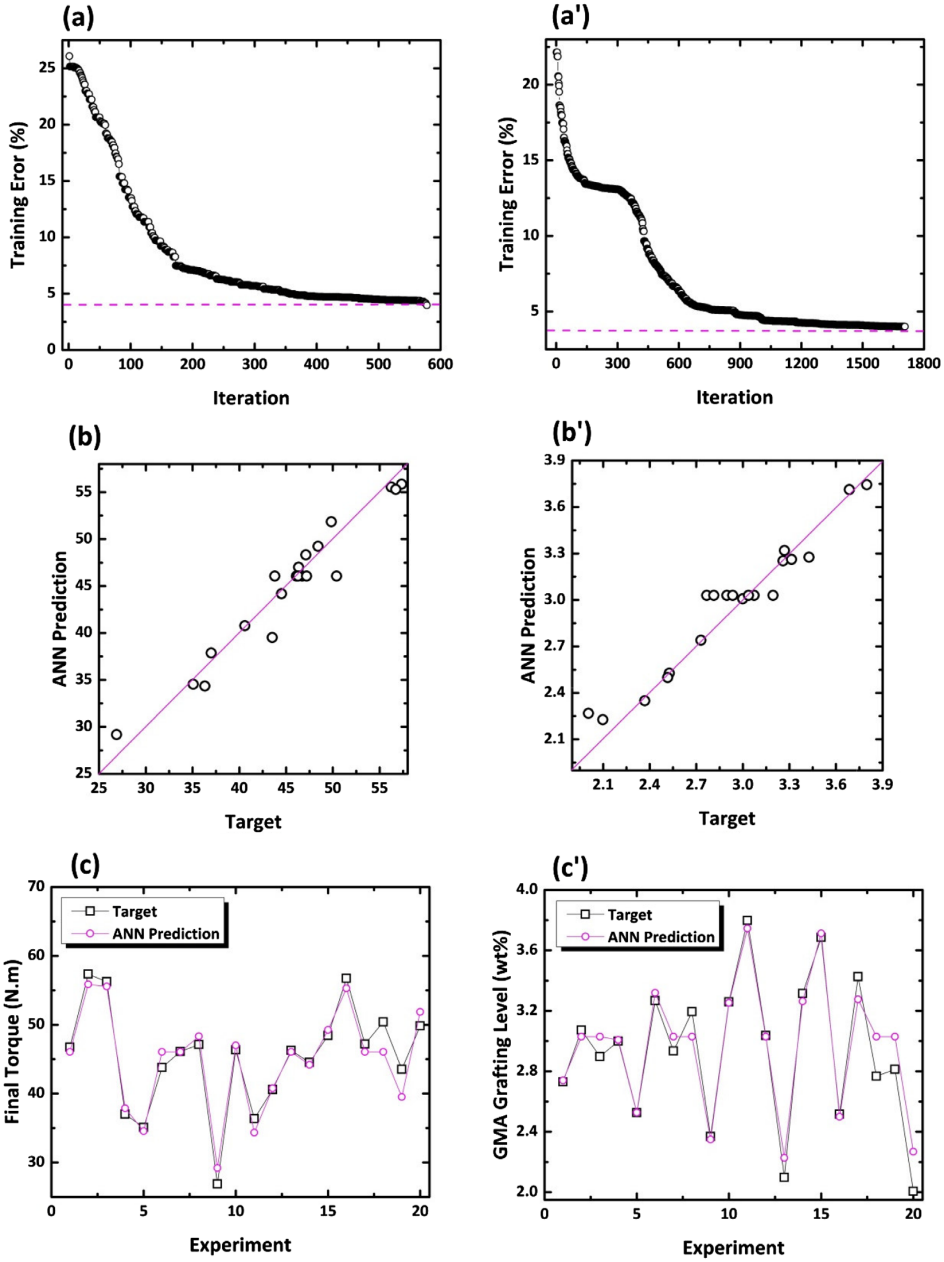
Iteration-dependent error variation of ANN model for optimization of final torque (a) and GMA grafting level (a′); Relationship between the experimental data and ANN outputs corresponding to final toque (b) and GMA grafting wt.% (b′); Comparison of experimental data with predicted values corresponding to training and test data sets for the final toque (c) and GMA grafting level (c′).

**Figure 6.**
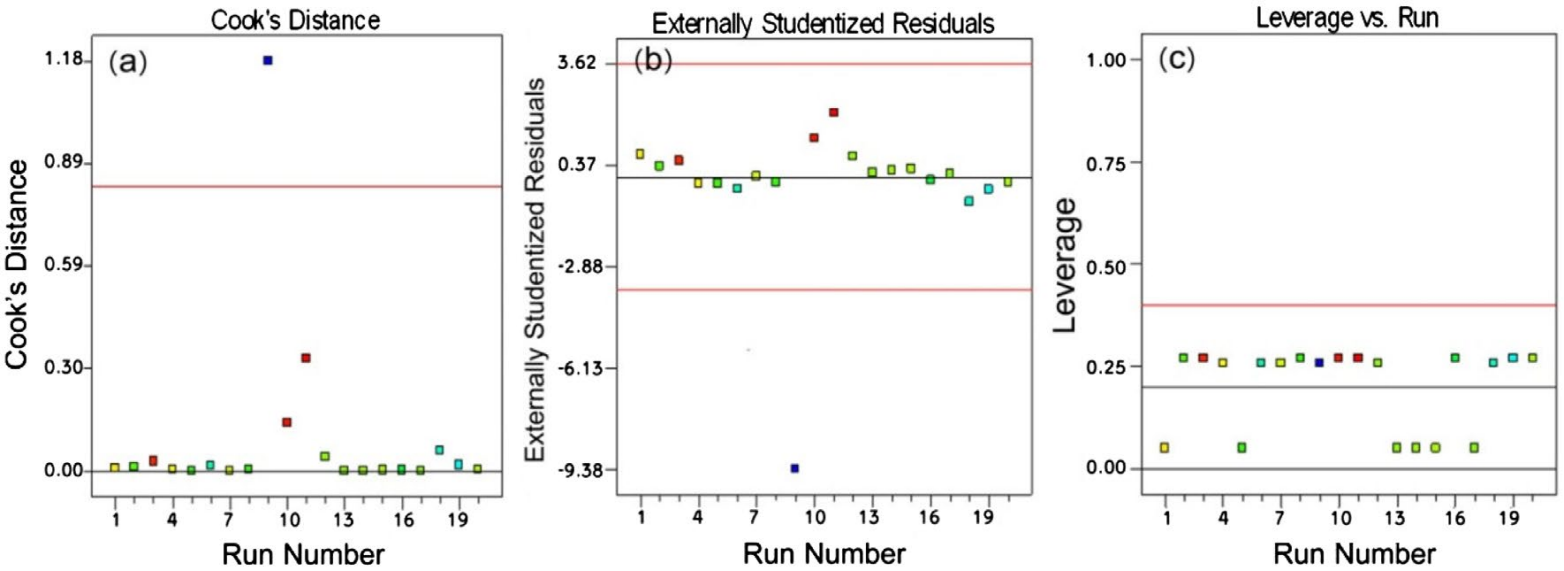
Quantities of different statistics for specifying unusual data: (a) Cook’s distance; (b) Externally studentized residuals; (c) Leverage vs. run**.**

**Figure 7.**
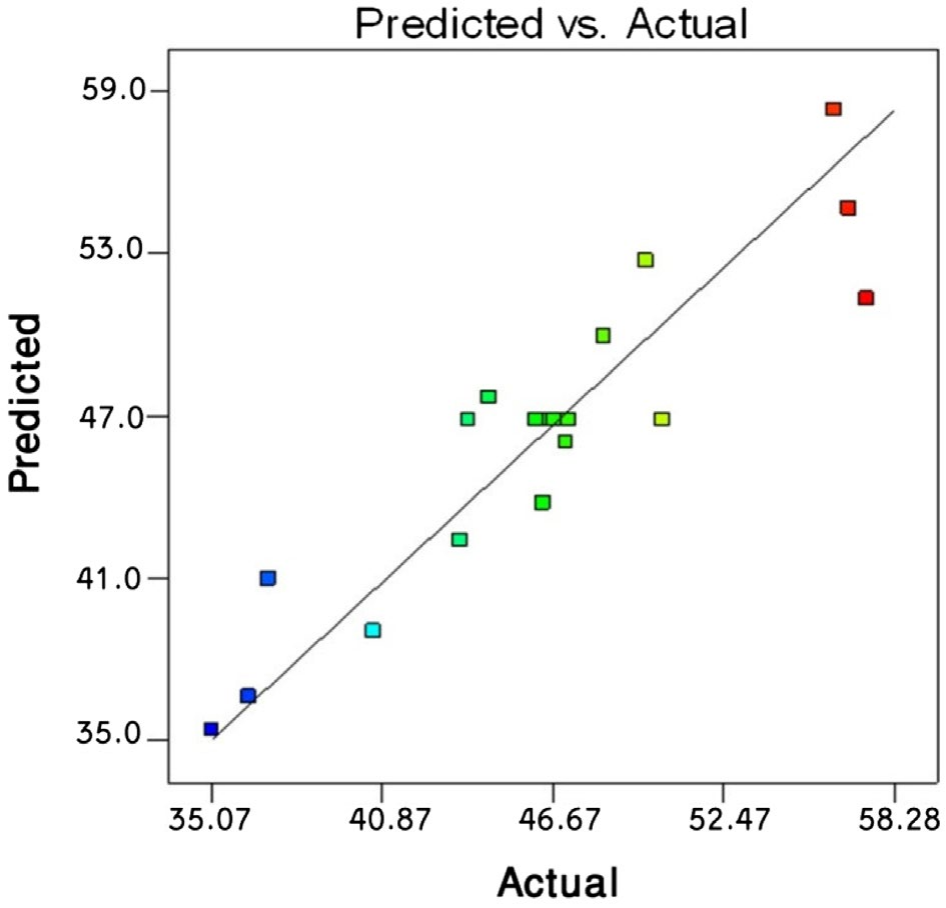
Predicted values of final torque (Nm) vs. actual values obtained using linear interpolating function yielded from RSM.

**Figure 8.**
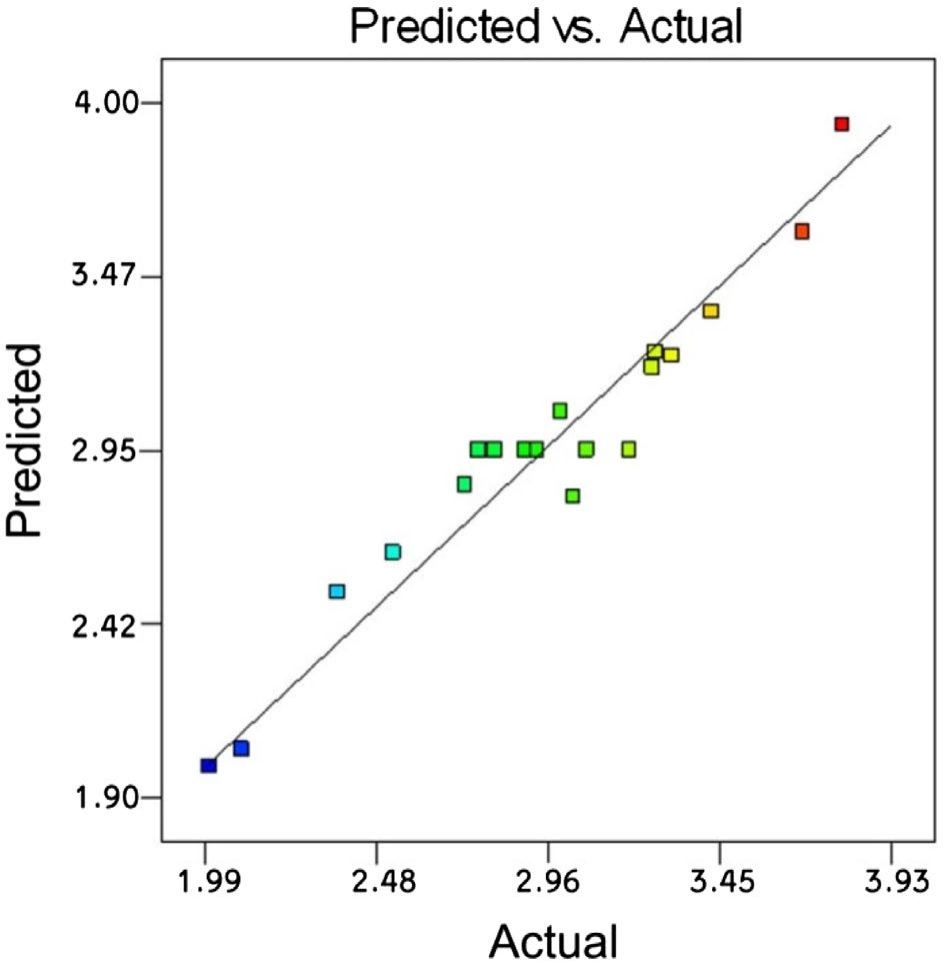
Predicted values of GMA grafting level (wt.%) vs. actual values obtained through linear interpolating function yielded from RSM.

**Figure 9.**
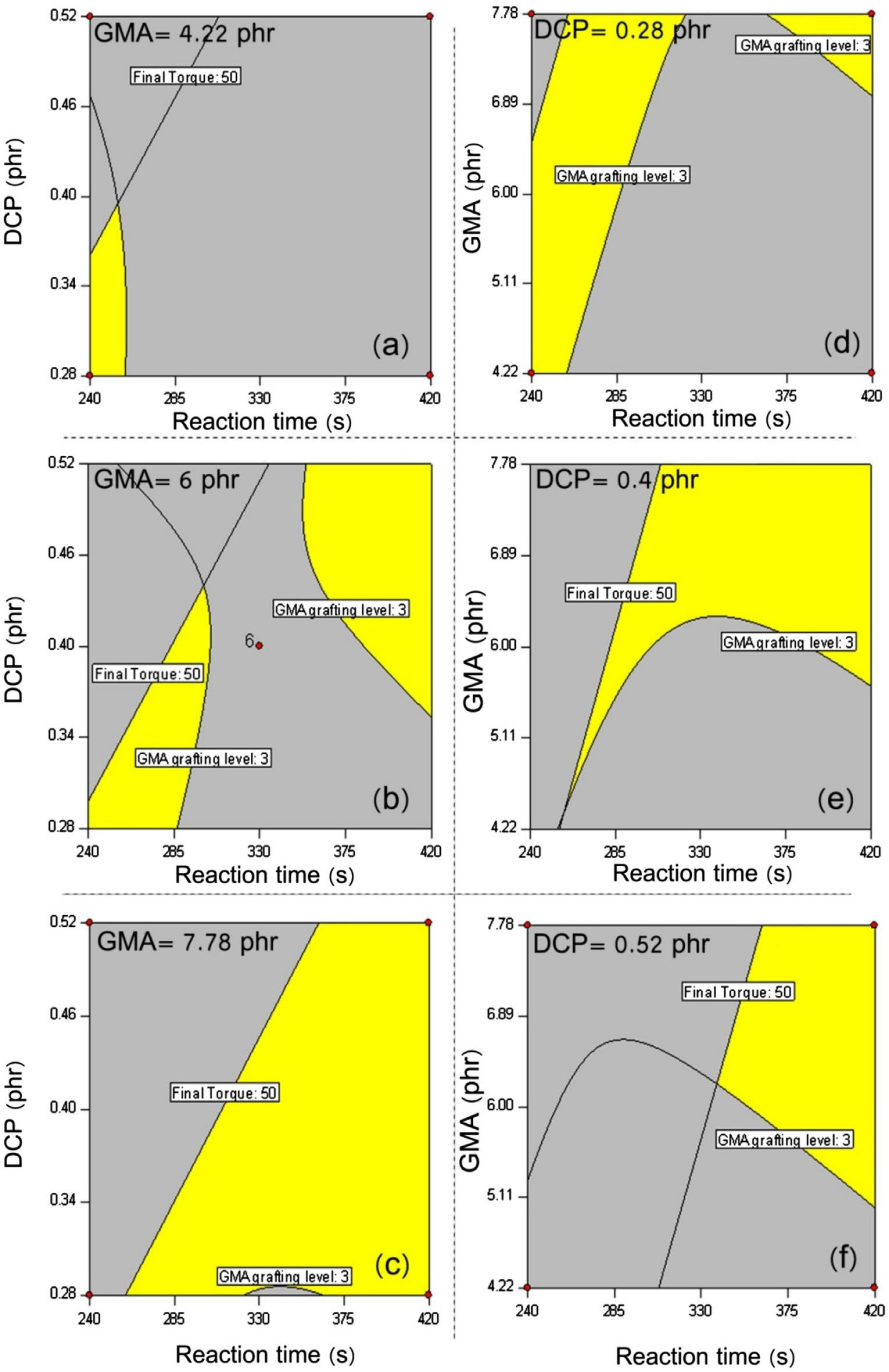
Overlaid plots illustrating the optimized regions yielded at constant GMA levels of 4.22 (a), 6.00 (b), and 7.78 (c) phr and constant DCP contents of 0.28 (d), 0.4 (e) and 0.52 (f) to maintain the final torque (Nm) and GMA grafting level (wt.%) in the range of 30–50 and 3–4, respectively**.**

**Figure 10.**
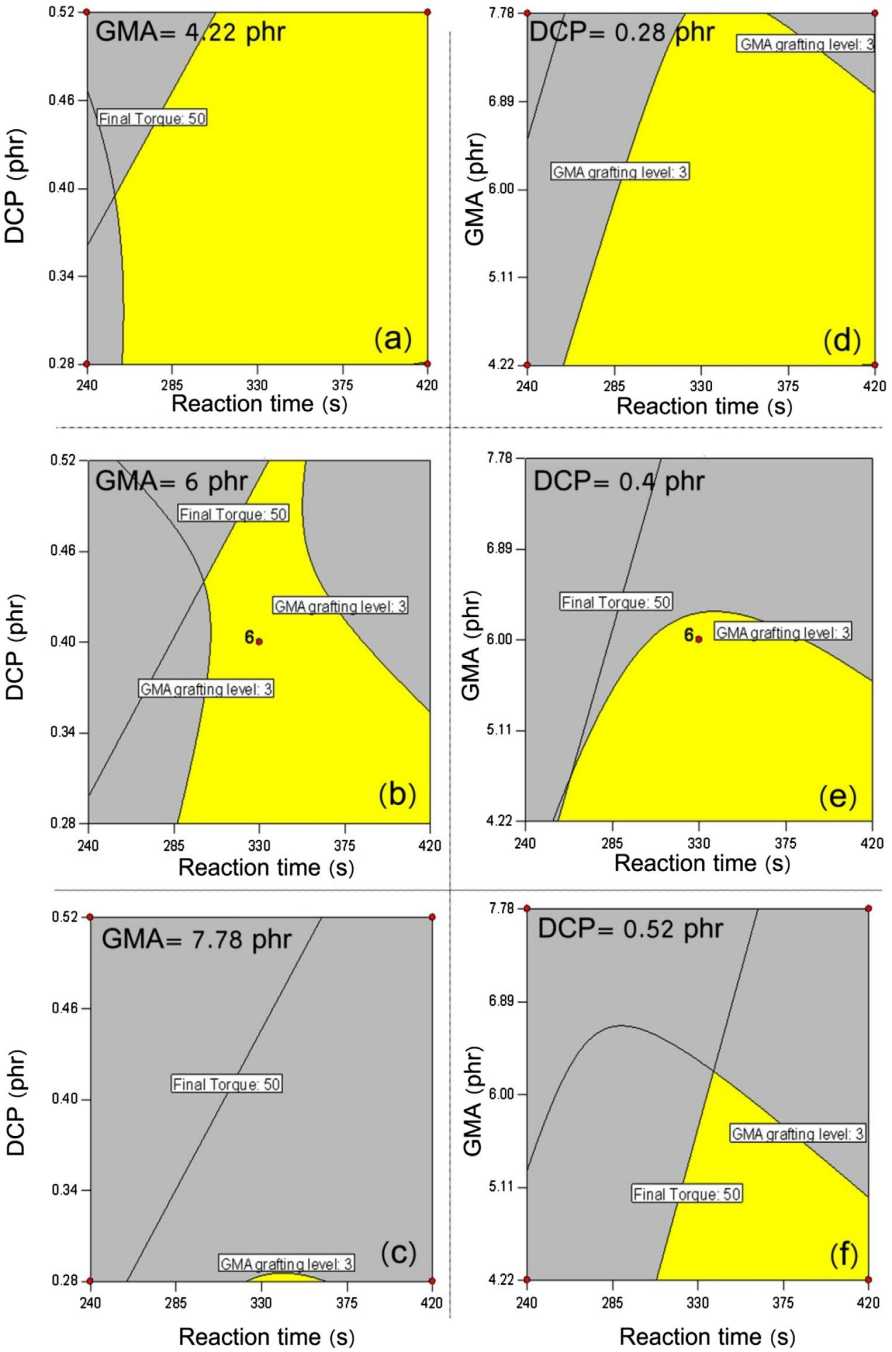
Overlaid plots illustrating the optimized regions yielded at constant GMA levels of 4.22 (a), 6.00 (b), and 7.78 (c) phr and constant DCP contents of 0.28 (d), 0.4 (e) and 0.52 (f) to maintain the final torque (Nm) and GMA grafting level (wt.%) in the range of 30–50 and 2–3, respectively**.**

**Figure 11.**
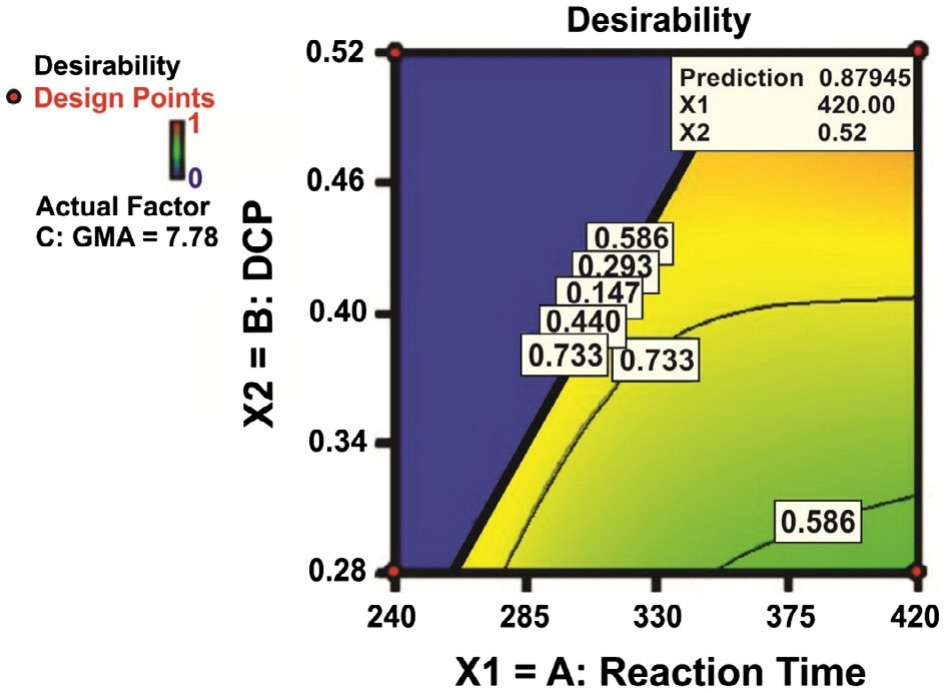
Contour plots of the desirability corresponding to simultaneous optimization of final torque and GMA grafting level**.**

## 4. Results and discussion

**Scheme 2.**
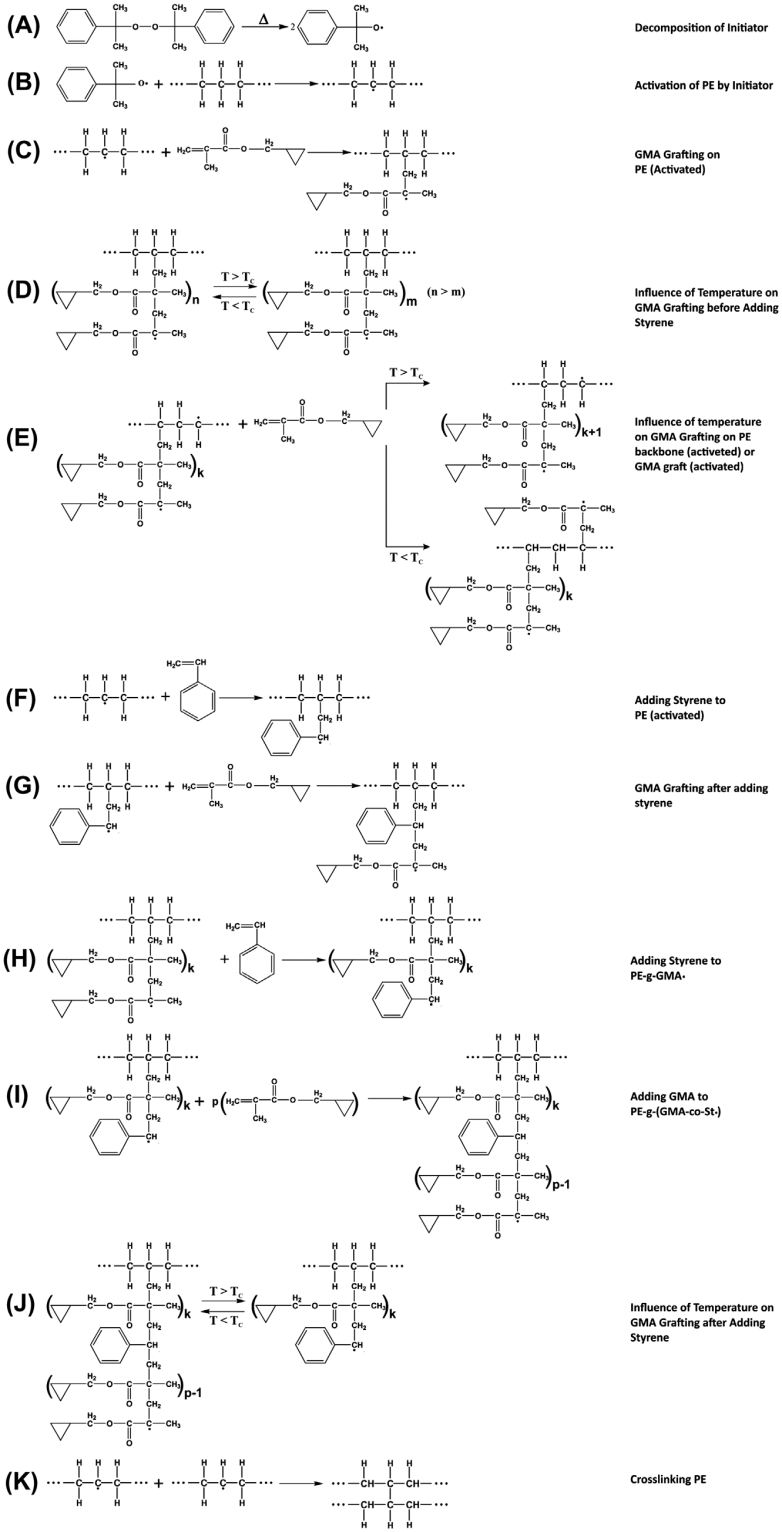
Possible reactions taking place during St-assisted GMA grafting on HDPE.

Studies reveal that melt free-radical grafting of GMA on HDPE is associated with by-side reactions, which are presented in Scheme 2.[17,20,22] Grafting takes place right after decomposition of DCP initiator. Li and Xie proved that homopolymerization of GMA is possible at concentrations above 8 wt.% and temperatures between 130 and 160 °C.[22] They also demonstrated occurrence of depolymerization of PGMA at temperatures above ceiling temperature (*T*_*C*_). Thus, GMA grafting is facilitated by the peroxide initiation, when temperature is below *T*_*C*_, while depolymerization of PGMA takes place until reaching equilibrium. In parallel, undesired cross-linking of HDPE chains takes place rapidly with the aid of free radicals in the system.

The competitive nature of cross-linking of HDPE and grafting of GMA on HDPE makes optimization of grafting difficult. This arises from considerable contribution of cross-linking to the final processing torque value recorded by the mixing machine. Optimization should be performed in a manner to enable distinguishing the contribution of GMA grafting to final torque, with comparatively lesser amount of energy needed to be overcome by mixer rotors.

To calculate GMA grafting ratio, samples with DCP content of 0.25 phr and variable GMA content of 2, 4, 6, and 8 phr were measured by FTIR and the calibration curve demonstrating the correlation between grafting ratio (%) and I_1730_/I_1368_ was obtained (Figure 4). This curve used for determination of grafting ratio of other samples prepared based on RSM (Table 2).

Evaluation of competence and/or adequacy of interpolating functions in view of coefficient of determination is well-established.[43,44] To disclose the competitive nature of desired GMA grafting on HDPE and unwelcomed cross-linking of HDPE in the presence of DCP, the capabilities of artificial intelligence and response surface design are blended. To meet this goal, a detailed and comprehensive data analyzing process was applied to the model system through which a global mathematical framework for studying functionalization of polyolefins with reactive monomers has been established. It was necessary to screen the experimental data to find the best interpolating function among the bewildering array of possibilities.

### 4.1. Modeling of grafting reaction using artificial intelligence approach

The alterations of MSEs of the best chromosome in training the experimental data of Table S2 are shown in Figure 5(a) and (a′). As in Figure 5(a) and (a′), the error in anticipating the final torque (GMA grafting degree) declined from 26.08% (22.14%) to 3.87% (3.99%) as the number of epochs increased to 578 (1707). In view of very low errors in calculations, it makes evident that the developed ANN model perceives the grafting behavior well.

Table 4 represents the results of modeling of final torque and grafting level as the target functions of this work. The desired level of satisfaction is featured in terms of the error criterion considered for training and test steps with MSE of 4 and 15%, respectively. For instance, in case of *y*_1_, totally 14 attempts were made to meet the essential error level. The first noticeable point here is the number of iterations allocated to the training procedure (successful at 14^th^ attempt) which springs from blending the ANN technique with GA that fulfills optimization. The outputs reveal that GA has repeated 578 times assigned to the evolutionary computations to learn successfully how to foresee the final torque through the developed network. For the mentioned case, the MSE values of the optimized network corresponding to training and test processes are calculated to be 6.3.9 × 10^−3^and 4.324 × 10^−2^, with errors of 3.97 and 10.39%, respectively. The second beneficial feature of this modeling is the ability to identify and report the scenario with maximum error in training and testing. Evidently, scenarios 9 and 1 revealed the maximum error of 7.56 and 14.23%, respectively. The coefficient of determination of 0.9435 between quantities of the target final torque and output of the optimized ANN is indicative of an almost successful modeling.

**Table 4.** Results related to ANN models' training and test steps.

Response	Final torque (*y*_1_)	GMA grafting (*y*_2_) (wt.%)
No. of attempts	14	30
No. of epochs	578	1707
Training MSE	6.309 × 10^−3^	6.396 × 10^−3^
Test MSE	4.324 × 10^−2^	6.467 × 10^−2^
Training error	3.9715%	3.9988%
Test error	10.3979%	12.7159%
Max. training error	7.557% (scenario 9)	9.153% (scenario 1)
Max. test error	14.236% (scenario 1)	14.675% (scenario 13)
*R*-squared	0.9435	0.9444
Correlation coefficient	0.9713	0.9718
Coefficient of efficiency	0.9414	0.9329
Goodness of fit (%)	75.794	74.109
Coefficient of determination	0.9435	0.9444

In a similar fashion, the training and testing of network for the prediction of GMA grafting level has been successfully performed, which put value on the truthfulness between learning and testing stages of the ANN. In case of GMA grafting percent, however, the accuracies are somewhat more dependent on the developed model.

To conclude, since model outputs agree well with the experimental results, the statistical quantities approve that the proposed model is answerable for the training and testing the experimental data on PO-*g*-MM preparation. Such degree of consistency makes the model an efficient tool for anticipating the grafting efficiency of GMA on HDPE in the internal mixer.

As can be seen in the next section, the adequacy of models predicting GMA grafting level will be improved by ignoring an unusual experimental data.

The performance of the developed ANN model has been further checked comparing the actual and predicted values of each response variable. From Figure 5(b) and (b′), errors in prediction of each response are reasonably small. Such meaningful similarities between the experimental and ANN model outputs replies on the accuracy of the ANN model in predicting the cases not directly being fed into the network during the training procedure. From the statistical standpoint, the slope and intercept of the regression equations for the outputs are very close to 1 and 0, respectively.

To give a brighter perspective of the accuracy of the optimized ANN developed in this work, the quantities of target functions for the whole trained and tested data are compared in Figure 5(c) and (c′). The plots prove that the optimized ANN predicts each response well. It is to be mentioned that alterations are plotted against experiment number, but they can easily be compared with the corresponding scenarios specified in Tables S2 and S3.

Tables S4–S7 in the SM list the weights and biases corresponding to the optimized final torque and GMA grafting level predicted through the ANN developed in this work. It is also worth mentioning that the mechanism of grafting that depends on the type of polyolefin and reactive monomer can seriously govern the authenticity of predictions.[45] Though ANN approach provides a good footprint of each response variable, but it suffers the inability to give information about the competitive nature of grafting and cross-linking reactions. This conclusion is in obvious disagreement with some previous studies that merely discuss on the reliability of predictions in view of *R* ^2^ quantities.[43] Since the complexity of problem dictates the level of confidence and authenticity, we further study this process through the RSM approach.

### 4.2. Optimization of GMA grafting efficiency based on RSM

#### 4.2.1. Identification of unusual data

The analysis of response surface designs normally involves three phases: (i) estimation of response function, (ii) model interpretation and visualization; (iii) identification of optimum operating conditions. From this perspective, different regression functions are examined to track, as far as possible, the undesired cross-linking reaction. For a standard CCD three types of regression functions are commonly being used including linear, linear with two factor interaction, and quadratic models. It is of vital importance to choice a model that guarantees the required significance and fits well the experimental data. This can be handled by computing the sequential sum of squares of each case and performing the lack of fits. The results based on Design Expert were indicative of two crucial features: The linear model provided the best regression function among studied models; the lack of fit tests reflected the significance of all models fitted to experimental data. Since the used models were found to be inadequate to fit the studied data-set, we decided to find and screen the unusual data. Taking into account the feeding sequence of ingredients into the reaction chamber, it can be realized that the Experiment 9 in Table 2 with *x*_1_ of 180 sec  should cogently have a very little chance to influence the reaction. This can be further approved taking a glimpse at the values of both responses corresponding to the aforementioned sample that stay far away from the range that other experimental runs fall within (Table 2). It should be mentioned here that RSM works on the basis of the polynomial approximation, while ANN takes the real correlation between variables into consideration to yield the best model with minimum error. Thus, such unusual datum in case of RSM is expected to insert a serious impact on the regression coefficients of the model and elimination of scenario 9 can prominently govern statistical evaluations. The statistical data known as Cook’s distance provides more insights into the identification of influential parameters. Figure 6 shows the statistics calculated for 20 experiments performed in this work. It can be realized from the Figure 6(a) that the statistic of the experiment nominated as Experiment 9, which is an axial point, stays far away from the others.

Such unexpected behavior typically arises from an uncommon predictor variable or inconsistencies in response variables at any particular level of changing variables, what can be realized plotting externally studentized and leverage plots (Figure 6(b) and (c)). The former tracks the out-of-range data, while the latter signifies the unusual quantities of predictor variables. The corresponding plots brightly witness the anomalous behaviors as the consequence of the assigned unusual response. In such circumstances, statistical interpretations should be revisited by eliminating unusual experimental data.

#### 4.2.2. Analysis of final torque

Once the unusual data was deleted from the analysis, it was attended to find the best reduced model in view of the adequacy. In a similar fashion to what performed in case of original data-set, approximation functions were examined and the linear model was appeared as the best among all possibilities in view of sequential sum of squares. Analogously, lack of fit test was indicative of adequacy of linear model in describing the behavior of final torque over the experimental range. Table 5 represents the ANOVA for the CCD linear model, in which the *p*-value of the linear model is very meaningful (<0.0001). In this table, all predictors are meaningful and the *p*-value of the lack of fit test (0.2260) replies on the adequacy of model. The *R*-squared value of 0.84 demonstrates that 84% of variability of the gathered data can be expressed by the linear model, which is practically acceptable.

**Table 5.** ANOVA table assigned to the response surface linear model relating to final torque (*y*_1_).

Source	Sum of squares	df	Mean square	*F* value	*p*-Value	
					Prob > *F*	
Model	611.46659	3	203.8222	26.4229572	< 0.0001	Significant
A-Reaction time	399.48184	1	399.48184	51.7877422	< 0.0001	
B-DCP	166.44643	1	166.44643	21.5776643	0.0003	
C-GMA	45.538326	1	45.538326	5.90346516	0.0281	
Residual	115.70745	15	7.7138299			
Lack of fit	92.780449	10	9.2780449	2.02338833	0.2260	Not Significant
Pure error	22.927	5	4.5854			
Cor. total	727.17404	18				
						
Std. dev.	2.7773782		*R*-squared	0.84088067		
Mean	46.293684		Adj. *R*-squared	0.8090568		
CV %	5.9994755		Pred. *R*-squared	0.71037297		
PRESS	210.60925		Adeq. precision	17.9436152		

The modified linear model applied to the real experimental data is given in Equation (5). This model can be employed to obtain main, interaction, contour and response surface plots to analyze this response further.

(5) $$y_{1}=51.468-0.068x_{1}+29.092x_{2}+1.026x_{3}$$

Similar to what discussed in case of ANN approach, the predictability of the reduced model toward final processing torque is illustrated (Figure 7). Although the ANN looks better than the RSM in view of *R* ^2^, the latter excludes the incorrect data.

#### 4.2.3. Analysis of GMA grafting level

Analysis of data based upon sequential sum of squares and lack of fit tests demonstrated that a quadratic model describes well the GMA grafting percent onto HDPE over the experimental test limits. Table 6 shows that the amount of GMA (parameter C) is meaningful and consistent with our expectations. Noticeably, the interaction terms between processing time (parameter A) and each of other two factors are significant, meanwhile A^2^ and C^2^ terms are meaningful on account of *p*-values given in Table 6.

**Table 6.** ANOVA table assigned to response surface quadratic model on GMA grafting level (*y*_2_).

Source	Sum of squares	df	Mean square	*F* value	*p*-Value	
					Prob > *F*	
Model	3.8652603	9	0.4294734	14.111326	0.0003	Significant
A-Reaction time	0.0801467	1	0.0801467	2.6334021	0.1391	
B-DCP	0.1005396	1	0.1005396	3.3034566	0.1025	
C-GMA	1.7392679	1	1.7392679	57.14761	<0.0001	
AB	0.5356125	1	0.5356125	17.598769	0.0023	
AC	0.574592	1	0.574592	18.879529	0.0019	
BC	0.003872	1	0.003872	0.1272234	0.7295	
A^2	0.7135138	1	0.7135138	23.444123	0.0009	
B^2	0.1370333	1	0.1370333	4.5025407	0.0628	
C^2	0.1561711	1	0.1561711	5.1313578	0.0497	
Residual	0.2739119	9	0.0304347			
Lack of fit	0.1440046	4	0.0360011	1.3856471	0.3585	Not Significant
Pure error	0.1299073	5	0.0259815			
Cor. total	4.1391722	18				
						
Std. dev.	0.1744553		*R*-squared	0.9338245		
Mean	2.9574842		Adj. *R*-squared	0.8676489		
CV %	5.8987742		Pred. *R*-squared	0.6037348		
PRESS	1.64021		Adeq. precision	15.319137		

The regression function with individual and interactive coefficients predicting the GMA grafting weigh percent is given by Equation (6). It can be concluded that final torque has not been governed by simultaneous change of chosen parameters, whereas interaction terms in the second-order model obtained for GMA grafting reveal a different manner. This contradictory behavior can be explained considering the fact that final processing torque receives both contributions from the grafting and cross-linking reactions.

(6) $$y_{2}=10.122-0.044x_{1}-2.255x_{2}+0.011x_{3}+0.024x_{1}x_{2}+0.002x_{1}x_{3}+0.103x_{2}x_{3}+3.6*10^{-5}x_{1}^{2}-6.942x_{2}^{2}-0.034x_{3}^{2}$$

The goodness of RSM in predicting the GMA grafting level is illustrated in Figure 8.

A comparison of Figures 7 and 8 suggests that the response surface design predicts the GMA grafting level better than that of final torque, what is further discussed and statistically analyzed later. This conclusion does not seem to hold true when looking at the ANN outputs. To shed more light on this criticism the sensitivity of responses to the explanatory variables was carefully revised and further discussed.

#### 4.2.4. Multi-objective optimization of final torque and GMA grafting level

There are three well-known routes for multi-objective optimization based upon RSM, among which the overlaid contour plots representation can be considered as the simplest as well as the most applicable representation of the best setting to simultaneously optimize multiple responses.[46] Fortunately, this approach in appropriate when we maximally have three predictor variables, which matches with the case we considered in this work. The second way is to keep one response as the target level and specify meaningful limitations for the alteration of the others. The concern here is to solve the problem with nonlinear regression methods. Others suggest simultaneous optimization of response variables by desirability functions. In this section, the overlaid plots and desirability functions are put into examination to give some new insights about the nature of competitive reactions. This situation is equivalent of reaching the highest GMA grafting, meanwhile very limited gelation arising from cross-linking reaction.

##### 4.2.4.1. Overlaid contour plots of final torque and GMA grafting level

From the physical point of view, it is crystal clear that the contribution of cross-linking by-side reactions is involved in the final processing torque quantities.[47,48] To make a deeper sense of the effect of such undesired reactions, it is essential to optimize simultaneously the GMA grafting level and final torque responses over the range of changing variables. The overlaid plots exhibiting variation pattern of the studied responses are illustrated through Figures 9 and 10. In virtue of previous experiences,[20] we fed to the software processing torque values in the range 30–50 Nm. It is obvious that such condition does not guarantee the governance of the main reaction, i.e. GMA grafting onto HDPE. To uncover this situation, two cases were considered at which GMA grafting level in wt% could take values in the range of 2–3 and 3–4, as in Figures 9 and 10, respectively. In these series of plots, the yellow region is illustrative of the area in which both responses are invited to take the aforementioned quantities. Also, the red dots are the cube points of the designed experiments. Figure 8 shows bivariate alteration of DCP content and reaction time to yield samples having GMA grafting level of 3–4 wt.% when final torque changes from 30 to 50 Nm. The comparison of overlaid plots of *a*, *b*, and *c* signifies that it is hardly imaginable to catch the optimized target when keeping the concentration of GMA low. From an optimistic standpoint, however, this might be possible at early stage of melt processing when a very low amount of DCP is used. By further increase of GMA content from 4.22 to 6 phr, when moving from plot *a* to *b*, the yellow region has been appeared at low DCP content and at the early stage of reaction. This is in excellent agreement with findings reported in our previous paper [20] in view of melt flow index measurements performed on some selected samples. The prolonged melt processing of POs in the presence of peroxide and sufficient amount of reactive monomer would cause destruction of gels, what speeds up the grafting reaction.[49] It agreement with this conclusion, as in plot *c*, higher concentrations of GMA together with prolonged processing time leads to appropriate GMA grafting onto HDPE irrespective of the DCP content. To further visualize the competitive nature of grafting and cross-linking reactions, one can compare overlaid plots obtained at different DCP contents (plots *d*, *e*, and *f* in Figure 9). Accordingly, when the concentration of DCP is low (plot *a*) it is possible to meet desired level of grating reaction even at early stages of the reaction, whereas higher concentrations of peroxide demand higher reaction times. To the best of the authors’ knowledge, such comprehensive perspective has not been represented and/or discussed yet. When the target grafting lies between 2 and 3 weight percent (Figure 10), increase of GMA concentration is of higher importance than the previous case, which is featured in very different overlaid plots of *a*, *b*, and *c* in Figure 10. Moreover, irrespective of DCP content, optimization of both responses needs more mixing time in the internal mixer and increase of DCP content hardens the possibility of grafting.

 To sum up, the diversity of optimization circumstances associated with multifarious overlaid plots reflected appropriately the sensitivity of GMA grafting efficiency to the chosen factors. In the following section, a broader picture of the influence of undesired cross-linking reaction has been provided, which brings advantageous features from engineering point of view.

##### 4.2.4.2. Desirability functions of final torque and GMA grafting level

Similar to what has been done in case of ANN modeling, response variables are converted to the desired variables taking values in the range of 0–1. In this regard, *y*_1_ and *y*_2_ are changed to *d*_1_ and *d*_2_ and explanatory variables took independent quantities to be correlated with the overall desirability defined as:

(7) $$D={\left(d_{1}\cdot d_{2}\right)}^{\frac{1}{2}}$$

The desirability is then interrelated to the structure of problem. Since the final processing torque was adjusted to take values in the range 30–50 Nm, this interval has the target desirability and the ones out of this range take zero value. The maximum desirability was set to take value of 50 Nm, hence, *d*_1_ can be defied as:

(8) $$d_{1}=\left\{\begin{array}{cc} 0; & y_{1}<30\\ \frac{y_{1}-30}{20}; & 30\leq y_{1}\leq 50\\ 0; & y_{1}>50 \end{array}\right.$$

For the second response variable, it would be preferred to have quantities between 2 and 4, hence, 4 takes the maximum desirability and quantities below 2 and above 4 have the minimum desirability set to zero. Thus, the desirability function corresponding to GMA grafting level is defined as:

(9) $$d_{2}=\left\{\begin{array}{cc} 0; & y_{1}<2\\ \frac{y_{2}-2}{2}; & 2\leq y_{1}\leq 4\\ 0; & y_{1}>4 \end{array}\right.$$

In this regard, the quantities of changing variables are manipulated to meet the highest D value, very close to unity. This has been done by Design Expert software. A number of the best solutions among the whole proposed by the software are listed in Table 7. As can be seen, the results are sorted in accord with desirability.

**Table 7.** Desirability values corresponding to the best cases of design with both responses being optimized.

Solutions: No.	Reaction time	DCP	GMA	Final torque	GMA grafting level	Desirability	
1	*420.00*	*0.52*	*7.78*	*46.01623*	*3.93165*	*0.879456*	*Selected*
2	419.31	0.52	7.78	46.06314	3.92403	0.87900	
3	420.00	0.52	7.78	45.89281	3.92581	0.87473	
4	420.00	0.52	7.72	45.95714	3.91770	0.87466	
5	420.00	0.52	7.69	45.89833	3.90926	0.87112	
6	376.42	0.52	7.78	48.98468	3.50667	0.84563	

To visualize the highest level of grafting as well as the lowest possible gelation, contour lines of the overall case are plotted in Figure 11, where GMA level is set to be 7.78 phr. The blue area in this figure signifies the situation with desirability of zero. This means that, for instance, the *y*_1_ takes values above 50 Nm and consequently desirability of *d*_1_ takes zero value. In this case, D takes zero value indicating that it is not possible to keep both responses simultaneously at their desired level. It can be realized from the figure that the reaction time plays a key role in controlling the grafting reaction. Moreover, to meet the optimum level of GMA grafting efficiency it is reasonable to keep the DCP content low enough and lengthen the time of reaction. From the perspective drawn, some new windows on the competitive nature of two parallel reactions of GMA grafting and HDPE cross-linking are opened. The developed mathematical approach in this work can be adapted to similar systems, thereby diversity of PO-*g*-MM compatibilizers can be designed and manufacture. The possibility of specifying situations at which the competitor reaction take place markedly gives more advantages to the current work.

## 4. Conclusion

Design and production of an appropriate compatibilizer for a polymer blend requires identification and fine-tuning of reaction conditions. Most of such precursors are prepared through free-radical grafting of a reactive monomer onto polyolefin backbone. To degree of connectivity of polymer components depends on whether or not the PO-*g*-MM strengthens the interface. The challenge in production of PO-*g*-MM compatibilizers, however, is that the main reaction of polymerization of monomers on the PO chains was found to be associated with some undesired reactions like cross-linking or chain secession. The literature lacks a clear basis for understanding the contribution of processing and material parameters to overall degree of grafting, what is typically featured by a rise in the processing torque monitored by the blending machine. In this work we combined the proficiencies of artificial intelligence and response surface methodologies to explore the best condition for the GMA grafting on HDPE with minimum possible cross-linking. The architecture of ANN has been optimized on the bedrock of GA. The *R*-squared values of around 95% witnessed that the optimized ANN model developed in this work properly captures the processing torque and GMA grafting level. This has been signaled by testing the untrained experimental data of aforementioned responses. With respect to accuracy of model, RSM ended in similar results, but after deleting the unusual observation, what realized from statistics of Cook’s distance, externally studentized residuals, and leverage vs. run criteria. In particular and for the first time, the impact of cross-linking on the processing torque was statistically studied. With the assistance of overlaid plots yielded from multi-objective optimization of processing torque and GMA grafting level, we examined and somehow identified the competitive nature of two possible reactions of grafting of GMA onto HDPE as well as unwelcome cross-linking of HDPE. It was found that harmonized manipulation of DCP content, GMA content, and reaction time brings about diversity of cases where both responses are at their desired levels. On the basis of desirability function concept, the optimized conditions for producing PO-*g*-MM with maximum GMA grafting was highlighted. This methodology brings new insights for designing and manufacturing compatibilizers through reactive blending of POs with polar monomers.

## Disclosure statement

No potential conflict of interest was reported by the authors.

## Supplemental data

Supplemental materials for this article can be accessed http://dx.doi.org/10.1080/15685551.2016.1239166.

## Supplementary Material

TDMP_1239166_Supplementary_Material.doc
