# Supplementary material for: Controlled grafting of vinylic monomers on polyolefins: a robust mathematical modeling approach
Source: Des Monomers Polym. 2016 Oct 25;20(1):250–68. doi: 10.1080/15685551.2016.1239166 (PMC5812188; doi:10.1080/15685551.2016.1239166)
Supplement: TDMP_1239166_Supplementary_Material.doc [file TDMP_A_1239166_SM2944.doc]

***Supporting Information to***

**Controlled Grafting of Vinylic Monomers on Polyolefins: A Robust Mathematical Modeling Approach**

**S1. Experimental design using RSM**

From the practical point of view, details on response surface design have been provided in a technically elaborated textbook by Montgomery who edges over the others working in the field of statistical design and quality control of systems [S1]. As he simply defines, “RSM is a collection of mathematical and statistical techniques useful for modeling and analysis of problems in applications where a response of interest is influenced by several variables and the objective is to optimize this response”. The excellence of RSM roots from its ability in providing a precise estimate of the model coefficients fitted on experimental data in a manner that a good pattern for the prediction of response is obtained. Corresponding to the defined parameters and responses, the response can be expressed as a function of changing variables by the following formula:


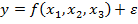
 (1)

in which the expected value of the response
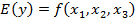
 is called a response surface.

Since most of times the relationship between the responding and changing variables is unknown, the first step in designing the problem is to find an interpolating function.

In the case that a linear approximation matches well with the experimental data, the following relation can be used:


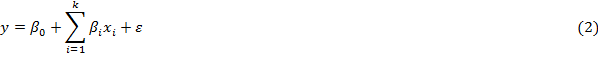


This model is very efficient when the design stays far away from optimum point. When the relationship between predictor and response variables has the curvature, polynomials with higher order like the second-order model are to be used. In opposition to the first-order approximations, these models are authentic near to optima.

The second-order model was shown to be sufficiently reliable in determining the curvature or interaction between the changing variables in the system:


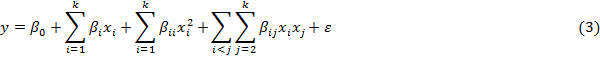


In the above expressions,
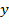
 is the predicted response
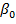
 is the intercept,
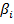
’s are the linear coefficients,
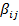
’s are the interactive coefficients,
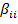
’s are the quadratic coefficients, and
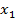
 ,
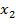
 and
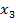
 are the coded independent variables, which are obtained in a manner that minimize the sum of squares of the model.

Central composite designs (CCDs) are a family of two-level factorial designs (or fractional factorial of resolution V) with carefully screened parameters for estimating the response surface of polynomial in the second-order model (equation 3).

**S2. Modeling by ANN approach**

The ANN was originally introduced inspiring from the biological neural network. Analogous to the neurons, the ANNs are networks with very large number of interconnected nodes, in which a node (neuron) is a simple processor that takes one (or more) inputs and produces an output. Each input into the node has an associated weight that determines the ‘‘intensity’’ of the input. Different processes are carried out by a node including the multiplication of inputs by their respective weights, the sum of the resultant numbers for all inputs and the determination of the output signal according to the result of this addition and an activation function, as can be seen in the equation 4:

| 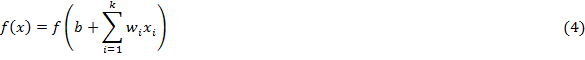 |  |
| --- | --- |

where
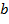
 is the bias of neurons,
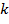
 is the number of elements and
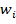
 is the weight of the input vector
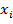
.

Basically, neural networks can be treated into two classes: feedforward networks and recurrent (or feedback) networks. The Multi-Layer Perceptron (MLP) is the commonly used feedforward neural network which consists of three or more layers, including an input layer, one or more hidden layers, and an output layer. A layer accepts all its inputs from either a preceding layer or the external world, but not from both. Similarly, a layer sends all its outputs to either a succeeding layer or the external world, but not to both. The input layer neurons receive data and the output neurons provide the ANN’s response to these input data. Intermediate layers, those that have no inputs or outputs to the external world, are called hidden layers and are part of the large internal pattern that determines a solution to the problem [S2].

These layers are parts of a large internal pattern that find a solution to the particular problem. In general, a basic three-layered MLP neural model is called a *p*–*q*–*r* neural model, where the variables, *p*, *q*, and *r*, are the total number of neurons in the input, hidden, and output layers, respectively. The values of *p* and *r* depend on the characteristics of the application and are determined precisely according to the input and output vector dimensions in a problem, respectively. On the other hand, the appropriate quantity of the hidden layer neurons (*q*) is mainly set by using trial and error methods [S3].

Each neuron in a specific layer is connected to the neurons of the previous layer. A neuron has two main components: (1) a weighted sum (
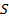
), which does a weighted sum of the inputs with components
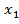
,
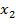
,
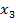
, …,
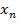
, i.e.
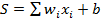
; and (2) a linear, nonlinear or logic function, which gives an output corresponding to
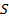
. The output from neuron
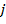
 in layer
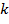
 is mostly calculated by the following equation:


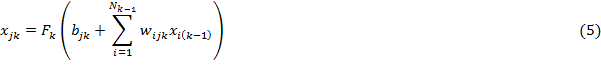


where the coefficient
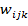
is the connection weight and
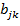
 is the bias of the network. They are the fitting parameters of the model and
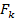
 is the transfer function of the layer
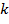
. The network output provided at the output layer has a pattern that is compared with the target vector by employing the Mean Squared Error (MSE) [S4]. The MSE of a neural network is defined as follows:


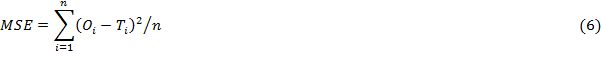


where
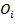
 is the desired output,
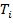
 is the network output, and
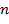
 is the number of data in the training data set or the cross validation data set.

**S3. Additional data on ANN modeling**

**Table S1.** Normalized data corresponding to input variables and target function.

| **Scenario** | **1^st^ Input**  **(**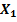**)**  **Reaction Time (sec)** | **2^nd^ Input**  **(**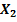**)**  **DCP**  **(phr)** | **3^rd^ Input**  **(**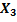**)**  **GMA**  **(phr)** | **1^st^ Output**  **(**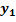**)**  **Final Torque (N.m)** | | **2^nd^ Output**  **(**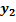**)**  **GMA Grafting Level (wt%)** |
| --- | --- | --- | --- | --- | --- | --- |
| **1** | 0 | 0 | 0 | | 0.54381 | 0.32589 |
| **2** | -0.6 | -0.6 | -0.59333 | | 0.15786 | 0.45982 |
| **3** | -0.6 | 0.6 | 0.59333 | | 0.92714 | 0.10826 |
| **4** | 0 | 1 | 0 | | 0.50706 | 0.14978 |
| **5** | 0 | 0 | 0 | | 0.10994 | 0.19085 |
| **6** | 0 | -1 | 0 | | -0.33508 | -0.59598 |
| **7** | 0 | 0 | 1 | | 0.41385 | 0.40848 |
| **8** | 0.6 | 0.6 | -0.59333 | | 0.09222 | -0.41964 |
| **9** | -1 | 0 | 0 | | -1 | -0.4308 |
| **10** | -0.6 | 0.6 | -0.59333 | | 0.95996 | -0.19196 |
| **11** | -0.6 | -0.6 | 0.59333 | | 1 | 0.58482 |
| **12** | 0 | 0 | -1 | | 0.27732 | -0.89732 |
| **13** | 0 | 0 | 0 | | 0.27273 | -0.15067 |
| **14** | 0 | 0 | 0 | | 0.30292 | -0.00558 |
| **15** | 0 | 0 | 0 | | 0.33377 | -0.09933 |
| **16** | 0.6 | -0.6 | 0.59333 | | -0.10075 | 0.39844 |
| **17** | 0 | 0 | 0 | | 0.26223 | 0.03571 |
| **18** | 1 | 0 | 0 | | -0.37972 | 0.875 |
| **19** | 0.6 | -0.6 | -0.59333 | | -0.46242 | -1 |
| **20** | 0.6 | 0.6 | 0.59333 | | 0.32852 | 1 |

**Table S2.** Data used for training of ANNs.

| **Experiment** | **Scenario** | **1^st^ Input**  **(**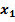**)**  **Reaction Time**  **(sec)** | **2^nd^ Input**  **(**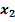**)**  **DCP**  **(phr)** | **3^rd^ Input**  **(**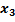**)**  **GMA**  **(phr)** | **1^st^ Output (**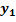**)**  **Final Torque (N.m)** | **2^nd^ Output (**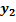**)**  **GMA GL (wt.%)** |
| --- | --- | --- | --- | --- | --- | --- |
| **1** | 14 | 330 | 0.4 | 6 | 46.73 | 2.73 |
| **2** | 11 | 240 | 0.28 | 7.78 | 57.35 | 3.073 |
| **3** | 3 | 240 | 0.52 | 7.78 | 56.24 | 2.897 |
| **4** | 6 | 330 | 0.2 | 6 | 37.01 | 2.999 |
| **5** | 19 | 420 | 0.28 | 4.22 | 35.07 | 2.526 |
| **6** | 5 | 330 | 0.4 | 6 | 43.79 | 3.268 |
| **7** | 17 | 330 | 0.4 | 6 | 46.11 | 2.934 |
| **8** | 20 | 420 | 0.52 | 7.78 | 47.12 | 3.194 |
| **9** | 9 | 180 | 0.4 | 6 | 26.88 | 2.368 |
| **10** | 12 | 330 | 0.4 | 3 | 46.34 | 3.259 |
| **11** | 18 | 480 | 0.4 | 6 | 36.33 | 3.798 |
| **12** | 16 | 420 | 0.28 | 7.78 | 40.58 | 3.0362 |
| **13** | 13 | 330 | 0.4 | 6 | 46.27 | 2.098 |
| **14** | 2 | 240 | 0.28 | 4.22 | 44.52 | 3.314 |
| **15** | 7 | 330 | 0.4 | 9 | 48.42 | 3.686 |
| **16** | 10 | 240 | 0.52 | 4.22 | 56.74 | 2.516 |

**Table S3.** Data used to test ANNs.

| **Experiment** | **Scenario** | **1^st^ Input**  **(**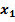**)**  **Reaction Time**  **(sec)** | **2^nd^ Input**  **(**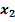**)**  **DCP**  **(phr)** | **3^rd^ Input**  **(**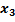**)**  **GMA**  **(phr)** | **1^st^ Output**  **(**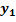**)**  **Final Torque (N.m)** | **2^nd^ Output**  **(**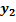**)**  **GMA GL (wt.%)** |
| --- | --- | --- | --- | --- | --- | --- |
| **17** | 15 | 330 | 0.4 | 6 | 47.2 | 3.426 |
| **18** | 1 | 330 | 0.4 | 6 | 50.4 | 2.767 |
| **19** | 8 | 420 | 0.52 | 4.22 | 43.52 | 2.813 |
| **20** | 4 | 330 | 0.6 | 6 | 49.84 | 2.006 |

**Table S4.** Optimal weights calculated for ANN related to the target function, final torque.

| **Input Layer &**  **1^st^ Hidden Layer** | **1^st^ Hidden Layer &**  **2^nd^ Hidden Layer** | **2^nd^ Hidden Layer &**  **3^rd^ Hidden Layer** | **3^rd^ Hidden Layer &**  **4^th^ Hidden Layer** | **4^th^ Hidden Layer &**  **5^th^ Hidden Layer** | **5^th^ Hidden Layer &**  **6^th^ Hidden Layer** | **6^th^ Hidden Layer &**  **7^th^ Hidden Layer** | **7^th^ Hidden Layer &**  **8^th^ Hidden Layer** | **8^th^ Hidden Layer &**  **Output Layer** |
| --- | --- | --- | --- | --- | --- | --- | --- | --- |
| -0.87524 | -0.91004 | -0.10737 | 0.1797 | -0.11244 | -0.65786 | -0.01906 | 0.96057 | -0.6489 |
| 0.55253 | -0.67389 | 0.72607 | 0.14281 | -0.58627 | 0.5959 | 0.45289 | -0.13952 | -0.99676 |
| -0.12037 | 0.82328 | 0.48103 | -0.63831 | -0.05674 | -0.47688 | -0.73226 | 0.09179 |  |
| 0.93131 | -0.06395 | -0.98365 | 0.49107 | 0.52463 | -0.35142 | 0.85543 | -0.83184 |  |
| 0.38088 | -0.12281 | -0.13444 | 0.11698 | -0.52788 | -0.69047 | 0.09706 | 0.89461 |  |
| -0.76589 | -0.42555 | 0.85459 | -0.04323 | 0.71902 | 0.71185 | 0.20781 | -0.5377 |  |
| 0.33557 | 0.28127 | 0.52395 | -0.72294 | -0.33778 | -0.99484 | 0.3901 | 0.75109 |  |
| -0.92812 | -0.49684 | 0.46625 | 0.60174 | -0.84324 | -0.97435 | 0.58225 | -0.85262 |  |
| -0.98871 | 0.90307 | 0.48014 | -0.12034 | -0.70801 | 0.41399 | -0.20071 |  |  |
| -0.57678 | 0.29327 | -0.79462 | 0.87299 | -0.76658 | -0.65084 | -0.95613 |  |  |
| 0.17691 | 0.19729 | -0.06603 | 0.7631 | 0.57472 | 0.98727 | -0.17596 |  |  |
| 0.78373 | -0.69894 | -0.5889 | 0.44141 | -0.19145 | 0.84124 | -0.48668 |  |  |
| 0.78715 | 0.36978 | -0.75608 | -0.73991 | -0.61442 | 0.15734 | -0.04941 |  |  |
| 0.90819 | 0.37093 | -0.89628 | 0.67652 | 0.51666 | 0.35065 | -0.70731 |  |  |
| -0.48786 | -0.69469 | 0.62041 | -0.67752 | -0.62974 | -0.65581 | -0.90568 |  |  |
| -0.61824 | 0.62751 | -0.25073 |  | -0.19637 | 0.62538 | 0.91813 |  |  |
| -0.04367 | -0.82269 | -0.91791 |  | 0.2853 | 0.3629 | -0.01521 |  |  |
| 0.20375 | 0.9908 | 0.89303 |  | 0.73536 | 0.80722 | -0.00123 |  |  |
| -0.77701 | 0.6991 | 0.55803 |  | -0.93103 | 0.88101 | -0.01127 |  |  |
| -0.07849 | 0.88876 | 0.76313 |  | 0.63599 | 0.39339 | 0.70061 |  |  |
| 0.68715 | -0.569 | -0.9327 |  | -0.9129 | 0.42976 | 0.74782 |  |  |
| 0.39149 | -0.92658 | 0.97035 |  | -0.03743 | 0.73543 | -0.77427 |  |  |
| 0.69126 | 0.24748 | -0.41403 |  | -0.54192 | -0.1739 | -0.618 |  |  |
| -0.08193 | 0.99292 | -0.3547 |  | -0.21566 | -0.26543 | -0.69153 |  |  |
| -0.53166 | 0.56944 | -0.14185 |  |  | -0.20803 |  |  |  |
| -0.94111 | 0.76652 | 0.24854 |  |  | 0.17188 |  |  |  |
| -0.93605 | -0.30139 | 0.54416 |  |  | 0.23267 |  |  |  |
|  | 0.15515 | -0.27964 |  |  | -0.33451 |  |  |  |
|  | 0.51359 | -0.94858 |  |  | 0.49384 |  |  |  |
|  | 0.66068 | -0.41306 |  |  | 0.24571 |  |  |  |
|  | -0.9035 | -0.98802 |  |  | -0.80147 |  |  |  |
|  | 0.35092 | 0.45612 |  |  | -0.82128 |  |  |  |
|  | 0.07126 | 0.74087 |  |  | 0.51759 |  |  |  |
|  | 0.07904 | 0.99717 |  |  | -0.68929 |  |  |  |
|  | -0.89961 | 0.70631 |  |  | -0.41267 |  |  |  |
|  | -0.33343 |  |  |  | -0.84817 |  |  |  |
|  | -0.59368 |  |  |  | 0.66507 |  |  |  |
|  | -0.49666 |  |  |  | 0.30254 |  |  |  |
|  | -0.40248 |  |  |  | -0.9899 |  |  |  |
|  | -0.60167 |  |  |  | -0.3667 |  |  |  |
|  | 0.25462 |  |  |  | -0.2326 |  |  |  |
|  | -0.86269 |  |  |  | -0.93466 |  |  |  |
|  | -0.42201 |  |  |  | 0.1454 |  |  |  |
|  | -0.65337 |  |  |  | -0.95947 |  |  |  |
|  | -0.4593 |  |  |  | 0.44691 |  |  |  |
|  | -0.70635 |  |  |  | -0.58483 |  |  |  |
|  | -0.02897 |  |  |  | -0.19229 |  |  |  |
|  | -0.264 |  |  |  | 0.72948 |  |  |  |
|  | -0.2714 |  |  |  |  |  |  |  |
|  | -0.81014 |  |  |  |  |  |  |  |
|  | -0.01594 |  |  |  |  |  |  |  |
|  | -0.20812 |  |  |  |  |  |  |  |
|  | 0.51728 |  |  |  |  |  |  |  |
|  | 0.93191 |  |  |  |  |  |  |  |
|  | 0.94466 |  |  |  |  |  |  |  |
|  | 0.70089 |  |  |  |  |  |  |  |
|  | 0.14008 |  |  |  |  |  |  |  |
|  | 0.12618 |  |  |  |  |  |  |  |
|  | 0.22552 |  |  |  |  |  |  |  |
|  | -0.54315 |  |  |  |  |  |  |  |
|  | -0.77535 |  |  |  |  |  |  |  |
|  | 0.6682 |  |  |  |  |  |  |  |
|  | 0.87292 |  |  |  |  |  |  |  |

**Table S5.** Optimal biases calculated for ANN related to the target function, final torque.

| **1^st^ Hidden Layer** | **2^nd^ Hidden Layer** | **3^rd^ Hidden Layer** | **4^th^ Hidden Layer** | **5^th^ Hidden Layer** | **6^th^ Hidden Layer** | **7^th^ Hidden Layer** | **8^th^ Hidden Layer** | **Output Layer** |
| --- | --- | --- | --- | --- | --- | --- | --- | --- |
| 0.13354 | 0.72005 | -0.69214 | -0.88814 | -0.89938 | 0.0333 | 0.79605 | -0.35373 | 0.15428 |
| 0.67309 | 0.887 | -0.16182 | 0.08805 | 0.87324 | 0.52267 | 0.47781 | -0.17771 |  |
| 0.89239 | -0.50622 | 0.4977 | -0.99517 | -0.24814 | -0.40342 | -0.01442 |  |  |
| -0.17752 | -0.49481 | 0.82193 |  | -0.50967 | -0.38225 | -0.96653 |  |  |
| 0.32847 | 0.37907 | 0.92966 |  | -0.63197 | 0.41013 |  |  |  |
| 0.91798 | -0.84012 |  |  | 0.37265 | -0.90315 |  |  |  |
| -0.65212 | -0.48535 |  |  | 0.91062 |  |  |  |  |
| 0.92168 |  |  |  | -0.81022 |  |  |  |  |
| 0.72831 |  |  |  |  |  |  |  |  |

**Table S6.** Optimal weights calculated for ANN related to the target function, grafting level.

| **Input Layer &**  **1^st^ Hidden Layer** | **1^st^ Hidden Layer &**  **2^nd^ Hidden Layer** | **2^nd^ Hidden Layer &**  **3^rd^ Hidden Layer** | **3^rd^ Hidden Layer &**  **4^th^ Hidden Layer** | **4^th^ Hidden Layer &**  **5^th^ Hidden Layer** | **5^th^ Hidden Layer &**  **6^th^ Hidden Layer** | **6^th^ Hidden Layer &**  **7^th^ Hidden Layer** | **7^th^ Hidden Layer &**  **8^th^ Hidden Layer** | **8^th^ Hidden Layer &**  **Output Layer** |
| --- | --- | --- | --- | --- | --- | --- | --- | --- |
| 0.87725 | 0.9601 | 0.98697 | 0.90886 | -0.36502 | 0.98542 | 0.13887 | 0.40499 | -0.99899 |
| -0.4644 | -0.18751 | -0.46723 | -0.54776 | 0.29253 | 0.42092 | 0.56686 | -0.82856 | 0.54588 |
| 0.46176 | 0.92757 | 0.9153 | -0.23298 | -0.70306 | -0.79505 | 0.91199 | 0.85411 |  |
| -0.83703 | 0.75645 | 0.8762 | 0.02455 | -0.01284 | -0.49219 | -0.03904 | 0.04874 |  |
| 0.79504 | 0.26361 | -0.14701 | 0.63738 | 0.95844 | -0.66676 | 0.82909 | -0.38714 |  |
| -0.56553 | 0.23288 | 0.07156 | 0.06453 | 0.86562 | 0.98745 | 0.9169 | 0.83241 |  |
| 0.96419 | 0.06045 | 0.68095 | -0.10417 | -0.32068 | -0.26964 | 0.09629 | -0.34304 |  |
| -0.87781 | -0.05802 | -0.08687 | -0.05502 | -0.56532 | -0.58069 | -0.99358 | -0.64783 |  |
| -0.68126 | -0.78588 | -0.52762 | 0.54941 | -0.79404 | 0.01521 | 0.60103 |  |  |
| 0.32823 | -0.9795 | 0.63237 | -0.49518 | 0.70877 | 0.32817 | -0.93511 |  |  |
| -0.23519 | -0.44677 | -0.98773 | -0.58791 | -0.38932 | -0.24184 | -0.36027 |  |  |
| -0.20116 | -0.94662 | 0.6419 | -0.91095 | 0.93724 | -0.42145 | 0.94399 |  |  |
| 0.70399 | 0.34568 | -0.02013 | -0.62201 | -0.43636 | -0.58671 | -0.76801 |  |  |
| 0.66909 | 0.21312 | -0.11404 | -0.9008 | -0.72069 | 0.49077 | 0.80964 |  |  |
| -0.97209 | -0.69498 | 0.10011 | 0.56722 | -0.86033 | 0.97602 | 0.82851 |  |  |
| -0.30076 | 0.69189 | -0.5106 |  | -0.28488 | -0.98847 | -0.27231 |  |  |
| -0.38446 | 0.53927 | 0.75653 |  | -0.23771 | -0.28287 | 0.60104 |  |  |
| 0.60795 | 0.49337 | -0.31343 |  | -0.92844 | 0.35251 | -0.14209 |  |  |
| 0.64583 | 0.32462 | -0.65396 |  | 0.27508 | -0.87623 | 0.33472 |  |  |
| 0.89707 | 0.44072 | 0.77634 |  | 0.26779 | -0.49489 | 0.99419 |  |  |
| 0.9261 | 0.97003 | 0.1343 |  | 0.78145 | 0.40978 | -0.33621 |  |  |
| 0.28407 | 0.5838 | -0.69429 |  | -0.8881 | -0.71258 | 0.88907 |  |  |
| 0.60367 | 0.97556 | 0.73973 |  | -0.63223 | 0.96619 | -0.44962 |  |  |
| -0.32932 | -0.98449 | 0.59531 |  | 0.54262 | 0.9315 | -0.23456 |  |  |
| -0.5743 | 0.81138 | -0.5349 |  |  | 0.78928 |  |  |  |
| -0.59705 | -0.11589 | -0.49007 |  |  | 0.84451 |  |  |  |
| -0.88824 | -0.12739 | 0.72102 |  |  | -0.06405 |  |  |  |
|  | -0.03441 | -0.98775 |  |  | -0.68507 |  |  |  |
|  | 0.77083 | -0.49616 |  |  | -0.37498 |  |  |  |
|  | 0.80796 | 0.17974 |  |  | 0.74761 |  |  |  |
|  | 0.89176 | -0.2235 |  |  | -0.93019 |  |  |  |
|  | -0.13374 | -0.87387 |  |  | -0.57923 |  |  |  |
|  | 0.04963 | 0.18665 |  |  | -0.50697 |  |  |  |
|  | -0.26741 | 0.82037 |  |  | -0.17853 |  |  |  |
|  | -0.94249 | 0.88186 |  |  | -0.76186 |  |  |  |
|  | 0.36477 |  |  |  | 0.44732 |  |  |  |
|  | 0.98806 |  |  |  | -0.85526 |  |  |  |
|  | -0.54306 |  |  |  | -0.577 |  |  |  |
|  | 0.36324 |  |  |  | 0.83198 |  |  |  |
|  | 0.88082 |  |  |  | 0.5673 |  |  |  |
|  | 0.62139 |  |  |  | -0.94146 |  |  |  |
|  | -0.11984 |  |  |  | -0.04066 |  |  |  |
|  | -0.4221 |  |  |  | 0.59208 |  |  |  |
|  | 0.42992 |  |  |  | -0.83615 |  |  |  |
|  | 0.46888 |  |  |  | 0.01333 |  |  |  |
|  | 0.14603 |  |  |  | -0.98339 |  |  |  |
|  | -0.58646 |  |  |  | -0.24107 |  |  |  |
|  | 0.05543 |  |  |  | 0.13771 |  |  |  |
|  | 0.03036 |  |  |  |  |  |  |  |
|  | -0.28702 |  |  |  |  |  |  |  |
|  | 0.70031 |  |  |  |  |  |  |  |
|  | 0.89674 |  |  |  |  |  |  |  |
|  | 0.0792 |  |  |  |  |  |  |  |
|  | -0.47135 |  |  |  |  |  |  |  |
|  | 0.08747 |  |  |  |  |  |  |  |
|  | -0.27443 |  |  |  |  |  |  |  |
|  | 0.2339 |  |  |  |  |  |  |  |
|  | -0.04584 |  |  |  |  |  |  |  |
|  | 0.72631 |  |  |  |  |  |  |  |
|  | 0.4558 |  |  |  |  |  |  |  |
|  | 0.64692 |  |  |  |  |  |  |  |
|  | -0.86515 |  |  |  |  |  |  |  |
|  | -0.8564 |  |  |  |  |  |  |  |

**Table S7.** Optimal Biases calculated for ANN related to the target function, grafting level.

| **1^st^ Hidden Layer** | **2^nd^ Hidden Layer** | **3^rd^ Hidden Layer** | **4^th^ Hidden Layer** | **5^th^ Hidden Layer** | **6^th^ Hidden Layer** | **7^th^ Hidden Layer** | **8^th^ Hidden Layer** | **Output Layer** |
| --- | --- | --- | --- | --- | --- | --- | --- | --- |
| 0.46425 | -0.69677 | 0.47284 | 0.83209 | 0.95629 | 0.19648 | 0.29484 | -0.01472 | 0.43127 |
| -0.00659 | 0.96456 | -0.66426 | 0.3799 | 0.93777 | -0.21976 | 0.5643 | 0.07869 |  |
| 0.2145 | -0.55717 | -0.23332 | -0.64245 | 0.27612 | 0.87503 | 0.37861 |  |  |
| 0.06548 | 0.70227 | -0.68721 |  | -0.14636 | -0.51442 | -0.20185 |  |  |
| 0.2399 | 0.21039 | -0.74105 |  | 0.43841 | 0.88691 |  |  |  |
| 0.39327 | -0.42342 |  |  | 0.70514 | 0.93085 |  |  |  |
| -0.42152 | 0.5325 |  |  | -0.60714 |  |  |  |  |
| 0.93134 |  |  |  | 0.77434 |  |  |  |  |
| -0.21084 |  |  |  |  |  |  |  |  |

**References:**

[S1] D.C. Montgomery, Introduction to Statistical Quality Control, 6th ed., John Wiley & Sons, Inc., Hoboken, NJ, 2009.

[S2] J. Zou, Y. Han, S.S. So, Overview of artificial neural networks, in: D.J. Livingstone (Ed.) Methods in Molecular Biology, Humana Press, Totowa, NJ, USA, 2008, pp. 15–23.

[S3] Y. Xu, Y. Zhu, G. Xiao, C. Ma, Application of artificial neural networks to predict corrosion behavior of Ni–SiC composite coatings deposited by ultrasonic electrodeposition, Ceram. Int. 40 (2014) 5425–5430.

[S4] H. Kaydani, A. Mohebbi, A comparison study of using optimization algorithms and artificial neural networks for predicting permeability, Journal of Petroleum Science and Engineering 112 (2013) 17-23.
